# Supplementary material for: Proximity to Photosystem II is necessary for activation of Plastid Terminal Oxidase (PTOX) for photoprotection
Source: Nat Commun. 2024 Jan 4;15:287. doi: 10.1038/s41467-023-44454-x (PMC10767095; doi:10.1038/s41467-023-44454-x)
Supplement: Supplementary file 1 — Supplementary Information [file 41467_2023_44454_MOESM1_ESM.pdf]

Supporting Information for

Proximity to Photosystem II is necessary for activation of Plastid Terminal Oxidase (PTOX) for photoprotection.

Pablo Ignacio Calzadilla[1], Junliang Song[1], Patrick Gallois[2] and Giles Nicholas Johnson[1]*

[1]Department of Earth and Environmental Sciences, Faculty of Science and Engineering, University of Manchester, Manchester, United Kingdom. [2]Faculty of Biology, Medicine and Health, University of Manchester, Manchester, United Kingdom.

*Prof. Giles Nicholas Johnson.
Email: giles.johnson@manchester.ac.uk

This file includes:

Figures S1 to S21
Supplementary Tables 1 and 2
SI References

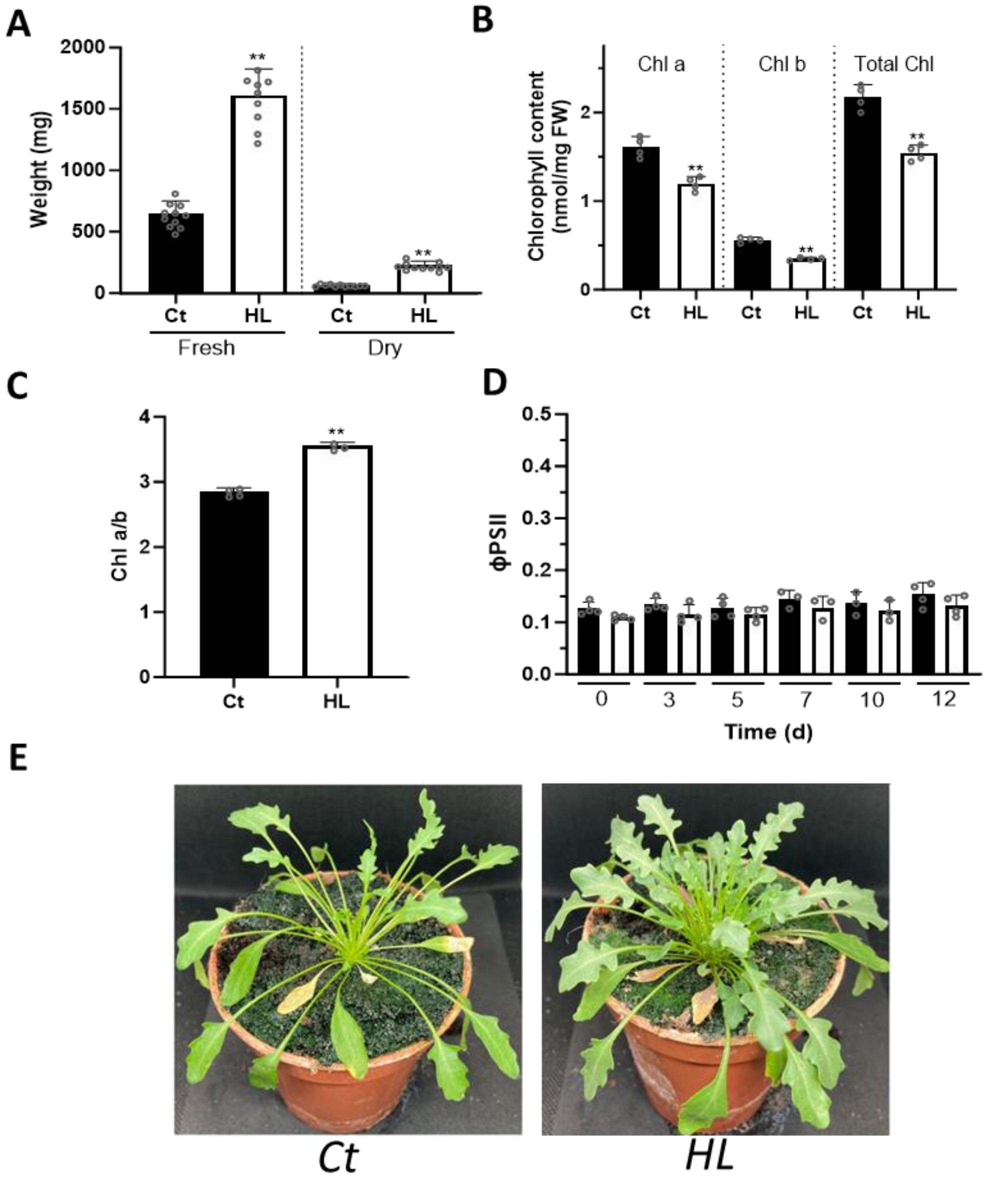

Figure S1. Characterization of high light acclimation in *Eutrema salsugineum* plants. A. Fresh and dry weight (mg, n = at least 9), and chlorophyll content (mmol.mg^{-1} FW, n = 4) of Eutrema plants subjected to control (Ct) or high light (HL) treatments for 10 d (**A** and **B**, respectively). **C.** Chlorophyll a/b ratio of Eutrema plants subjected to Ct and HL treatments for 10 d (n = 4). **D.** Quantum yield of PSII (ΦPSII) measured at 21% or 1% O_2 (black and white bars, respectively), during the Ct treatment (n = at least 3). **E.** Representative images of Eutrema plants at the end of the experiment. Data are the mean ± SD of independent biological replicates. The asterisk indicates differences between treatments (Student's test, $p < 0.01$, two-sided). All raw data, with number of replicates per data point, are provided in the source data file.

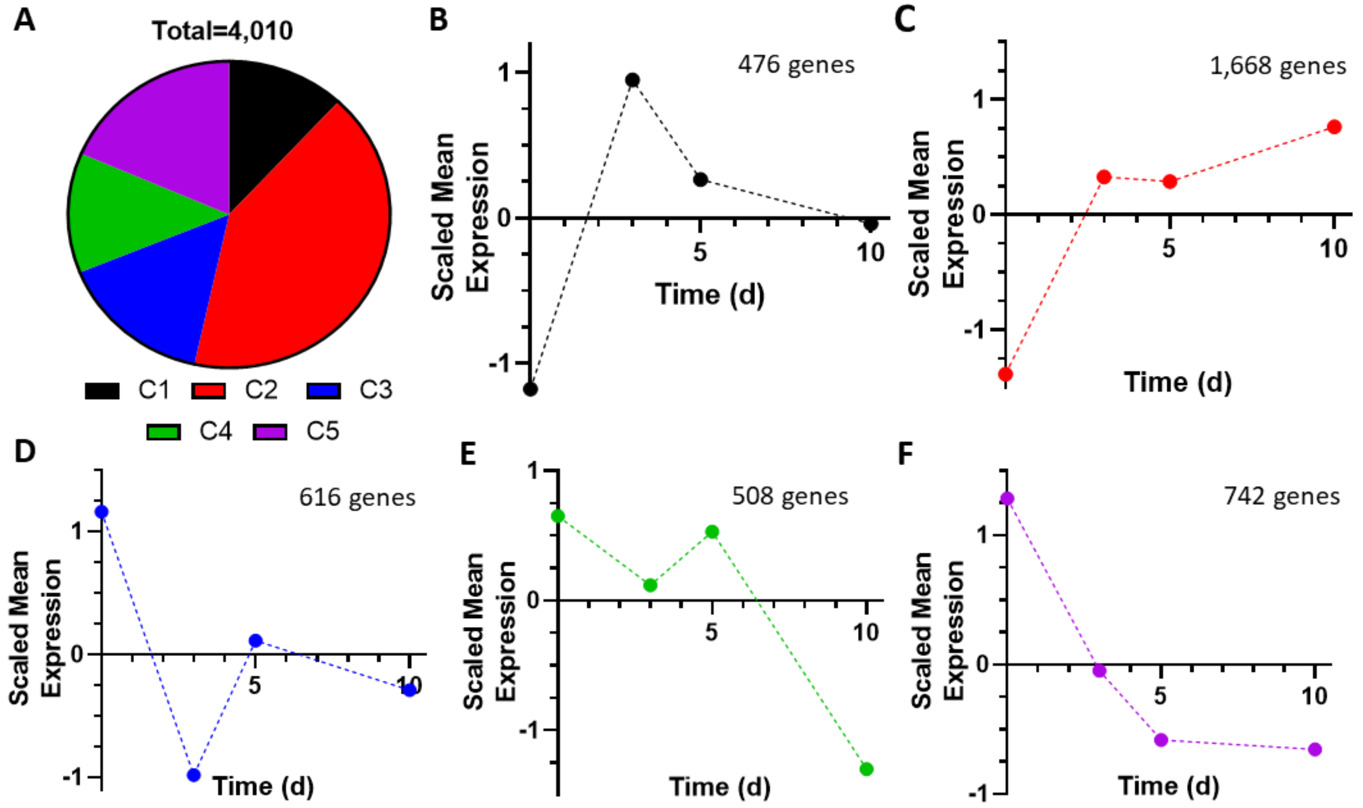

Figure S2. Gene expression profiles of the different clusters identified in the RNAseq analysis. A. A total of 4,010 differentially expressed genes were clustered in 5 different groups, each of them with a particular expression profile along the high light treatment. **B.** Expression profile of Cluster 1 (476 genes); **C.** Expression profile of Cluster 2 (1,668 genes); **D.** Expression profile of Cluster 3 (616 genes); **E.** Expression profile of Cluster 4 (508 genes); **F.** Expression profile of Cluster 5 (742 genes).

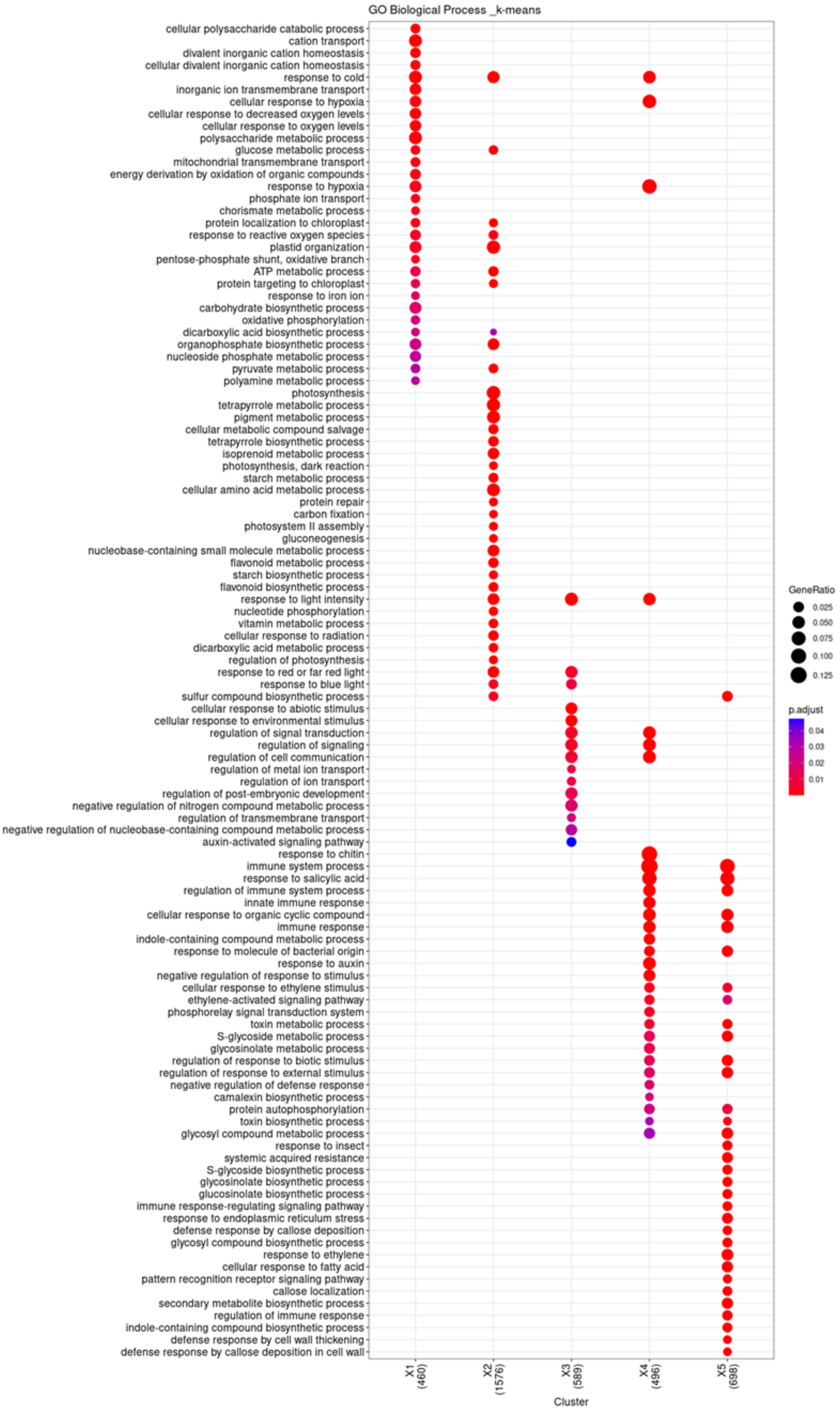

Figure S3. GO enrichment analysis of the clustered genes in the ‘Biological Process’ category. In brackets, the number of genes identified in this category for each cluster. Clusters 1 and 2 were mainly enriched in terms related to *photosynthesis*, *plastid organization* and *ROS responses*; while Clusters 3 to 5 to *hormone responses* and *signalling regulation*.

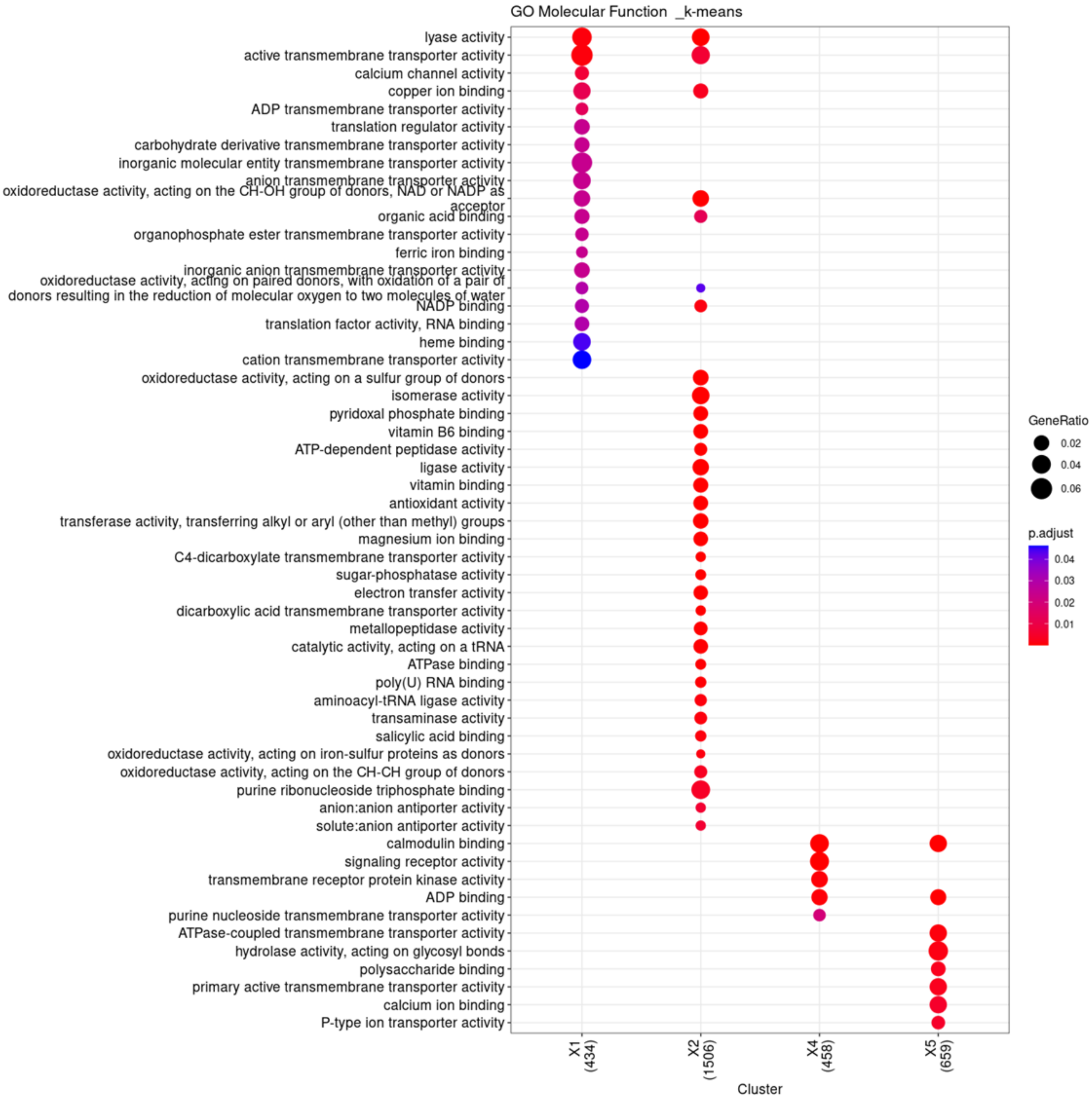

Figure S4. GO enrichment analysis of the clustered genes in the 'Molecular function' category. In brackets, the number of genes identified in this category for each cluster. Cluster 1 and 2 were enriched in several *oxidoreductase activity* and *transmembrane transporter activity* terms; while Cluster 4 and 5 in the latter and in *signalling translation* terms. No significant GO enrichment was observed for Cluster 3.

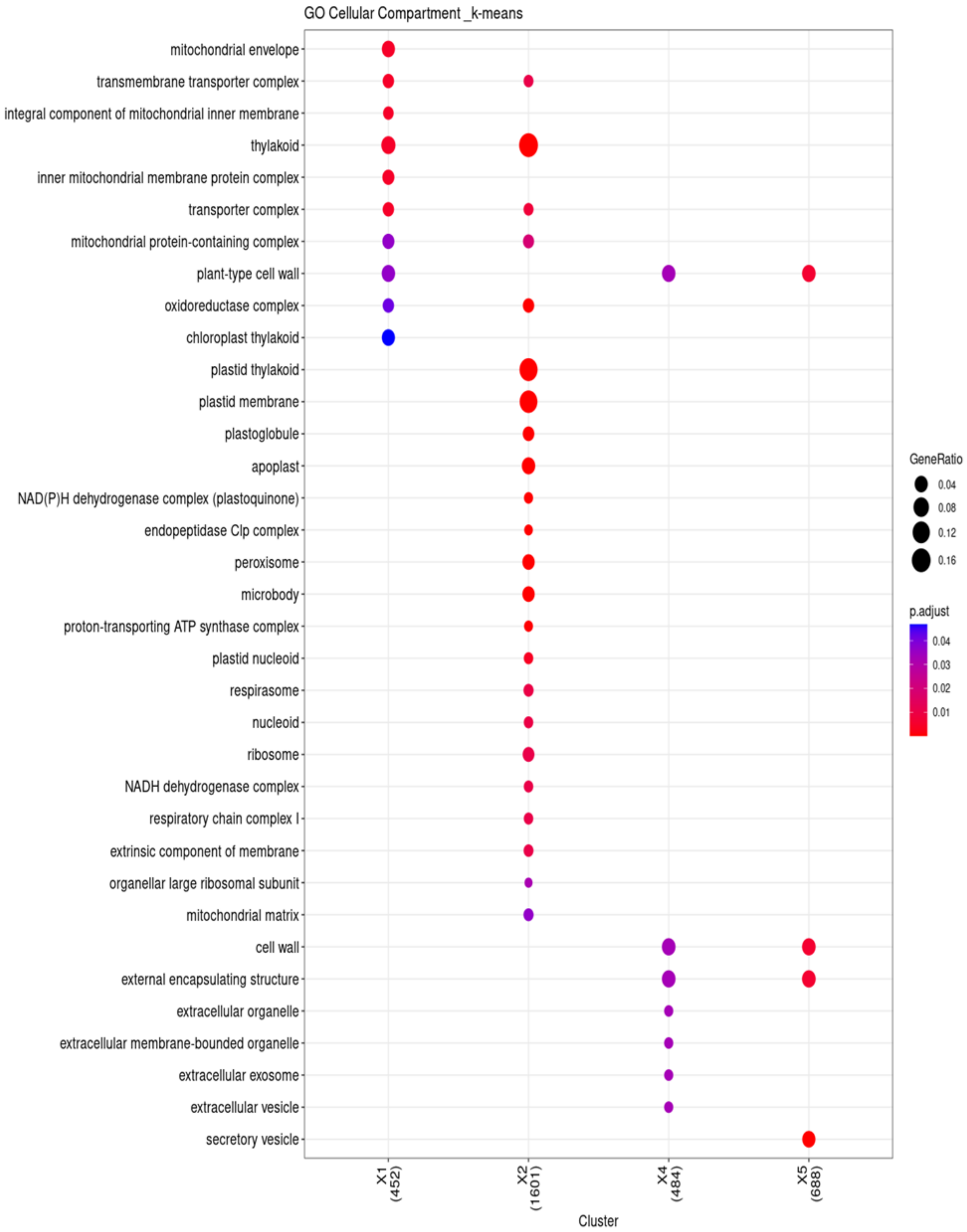

Figure S5. GO enrichment analysis of the clustered genes in the 'Cellular compartment' category. In brackets, the number of genes identified in this category for each cluster. Cluster 1 and 2 were enriched in *mitochondria* and *chloroplast thylakoids* related terms, being Cluster 2 more associated with the latest. By contrast, Cluster 4 and 5 were associated with *cell wall and extracellular components*. Cluster 3 shows no significant GO enrichment for the 'Cellular Compartment' group.

		Likelihood	
	**Predictor software**	**Chloroplast stroma**	**Thylakoid lumen**
**Eutrema PTOX**	TargetP -2.0	0.96	0.0027
	PredSL	0.90	-----
**Arabidopsis PTOX**	TargetP -2.0	0.95	0.011
	PredSL	0.98	-----
**OEC16**	TargetP -2.0	0.15	0.85
	PredSL	-----	0.99
**Plastocyanin**	TargetP -2.0	0.15	0.85
	PredSL	-----	0.99

Eutrema PTOX
>XP_006413676.1:6-346 ubiquinol oxidase 4, chloroplastic/chromoplastic [Eutrema salsugineum]
MAATVAISGISPRPLIALRRSRAAVSYSTSHRLLLHRPLSSPRLLFRNIHRVQATILQDDEEKVVVEESFKAETFPGKVPLEEPNMSSSTSALEAWIIKLEQGVNVFLTDSVIKILDTLYRDRTYP
RFFVLETIARVPYFAFMSVLHMYETFGWWRRADYLKVHFAESWNEMHHLLIMEELGGNSWWFDRFLAQHIATFYYFMTVFLYIISPRMAYHFSECVESHAYETYDKFLKASGEELKNSP
APDIAVKYYTGSDLYLFDEFQTARAPNTRRPTIENLYDVFVNIRDDEAEHCKTMRACQTLGSLRSPHSILEDDDCNEESGCVVPEAHCEGIVDCIKKSITN

Arabidopsis PTOX
>AT4G22260 IM Alternative oxidase family protein [Arabidopsis thaliana(thale cress)]
MAAISGISSGTLTISRPLVTLRRSRAAVSYSSSHRLLHHLPLSSRRLLLRNNHRVQATILQDDEEKVVVEESFKAETSTGTEPLEEPNMSSSSTSAFETWIIKLEQGVNVFLTDSVIKILDTLYRD
RTYARFFVLETIARVPYFAFMSVLHMYETFGWWRRADYLKVHFAESWNEMHHLLIMEELGGNSWWFDRFLAQHIATFYYFMTVFLYILSPRMAYHFSECVESHAYETYDKFLKASGEE
LKNMPAPDIAVKYYTGGDLYLFDEFQTSRTPNTRRPVIENLYDVFVNIRDDEAEHCKTMRACQTLGSLRSPHSILEDDDTEEESGCVVPEEAHCEGIVDCLKKSITS

TAT-translocated protein
>CAB40384.1 16 kDa polypeptide of oxygen-evolving complex [Arabidopsis thaliana]
MASMGGLHGASPAVLEGSLKINGSSRLNGSGRVAVAQRSRLVVRAQQSEETSRRSVIGLVAAGLAGGSFVQAVLADAISIKVGPPPAPSGGLPGTDNSDQARDFALALKDRFYLQPLPP
TEAAARAKESAKDIINVKPLIDRKAWPYVQNDLRSKASYLRYDLNTIISSKPKDEKKSLKDLTTKLFDTIDNKSPSQAEKYYAETVSALNEVLAKLG

SEC-translocated protein
>AAA32834.1 plastocyanin [Arabidopsis thaliana]
MAAITSATVTIPSFTGLKLAVSSKPKTLSTISRSSSATRAPPKLALKSSLKDFGVIAVATAASIVLAGNAMAMEVLLGSDDGSLAFVPSEFTVAKGEKIVFKNNAGFPHNVVFDEDEIPSGVD
ASKISMDETALLNGAGETYEVTLTEPGSYGFYCAPHQGAGMVGKLTVK

Figure S6. Prediction of PTOX chloroplast-localization based on its protein sequence. The subcellular localization of PTOX from *Eutrema salsugineum* and *Arabidopsis thaliana* was analysed using two different software predictors (TargetP-2.0[1] and PredSL[2]). As positive controls of thylakoid lumen-located proteins we used OEC16 and plastocyanin, translocated from the stroma to the lumen through the TAT and SEC pathway, respectively[3, 4].

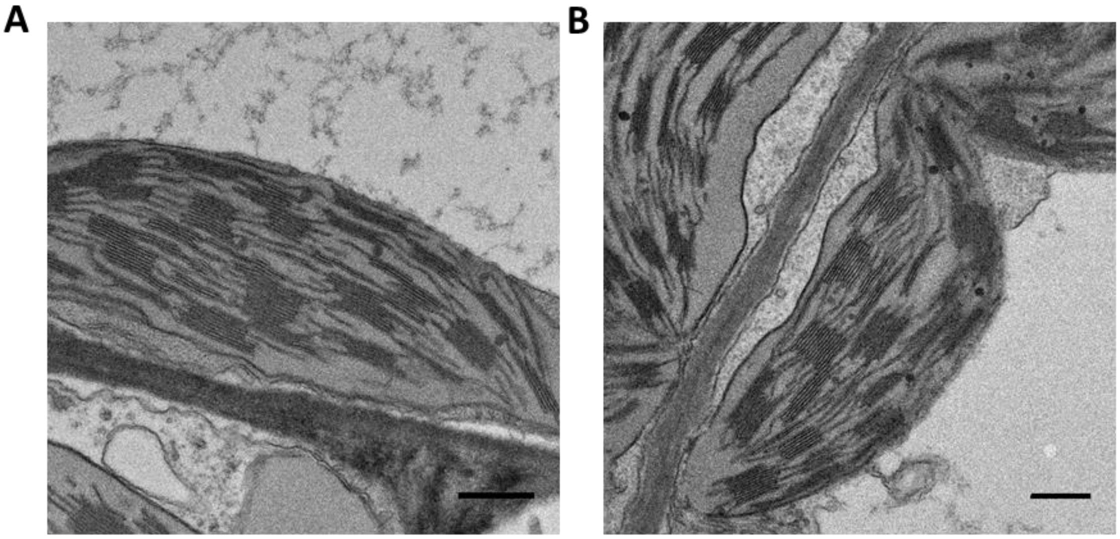

Figure S7. Chloroplast ultrastructure of *Eutrema salsugineum* plants at 0 (A) and 10 d (B) of the control treatment. Scale bars = 0.5 µm**.**

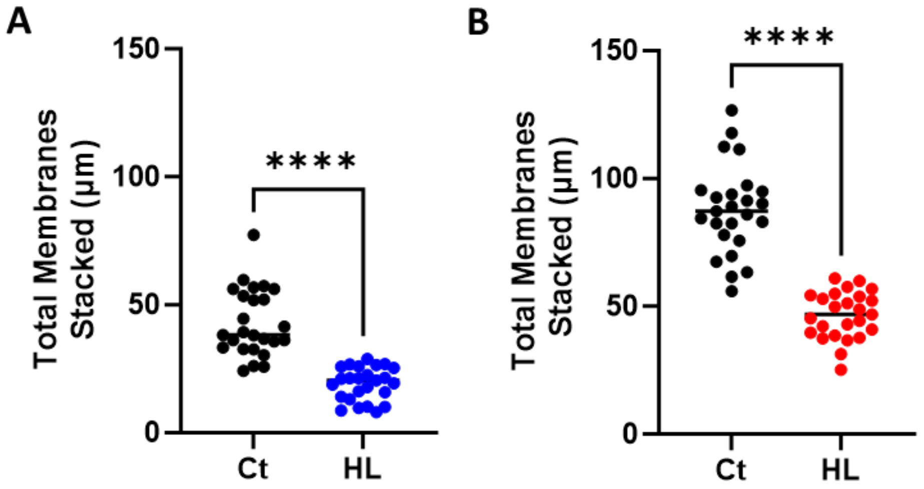

Figure S8. Measurements of the total length of thylakoid membranes appressed within grana stacks. The total length (µm) was measured in Eutrema (**A**) and Arabidopsis (**B**) wt plants. Ct, Control treatment; HL, High light-treated plants. Measurements were performed after 10 d of HL in Eutrema and 12 d of HL in Arabidopsis. At least 25 chloroplasts were measured in each condition. A minimum of 10 non-overlapping fields of view of the ultra-thin sections were examined, and leaf pieces of two plants were fixed for the analysis. The asterisk indicates differences between Ct and HL treatments (Welch's t-test, $p < 0.0001$, two-sided).

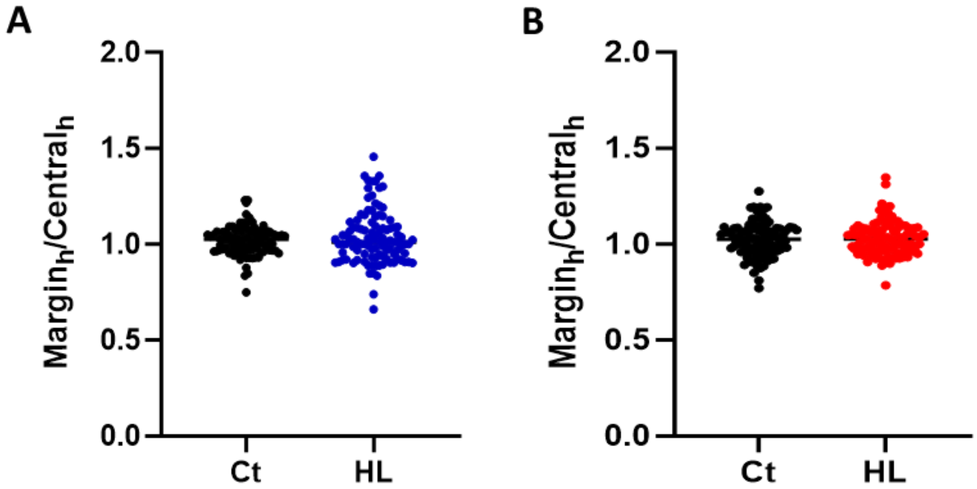

Figure S9. Measurements of the swelling of the thylakoid grana margins under stress conditions. The height of the central and marginal regions of the thylakoid membranes were measured, and the ratio of the latter with the former calculated in Eutrema (**A**) and Arabidopsis (**B**) wt plants. Ct, Control treatment; HL, High light-treated plants. Measurements were performed after 10 d of HL in Eutrema and 12 d of HL in Arabidopsis. At least 100 measurements were performed in each condition. A minimum of 25 non-overlapping fields of view of the ultra-thin sections were examined, and leaf pieces of two plants were fixed for the analysis.

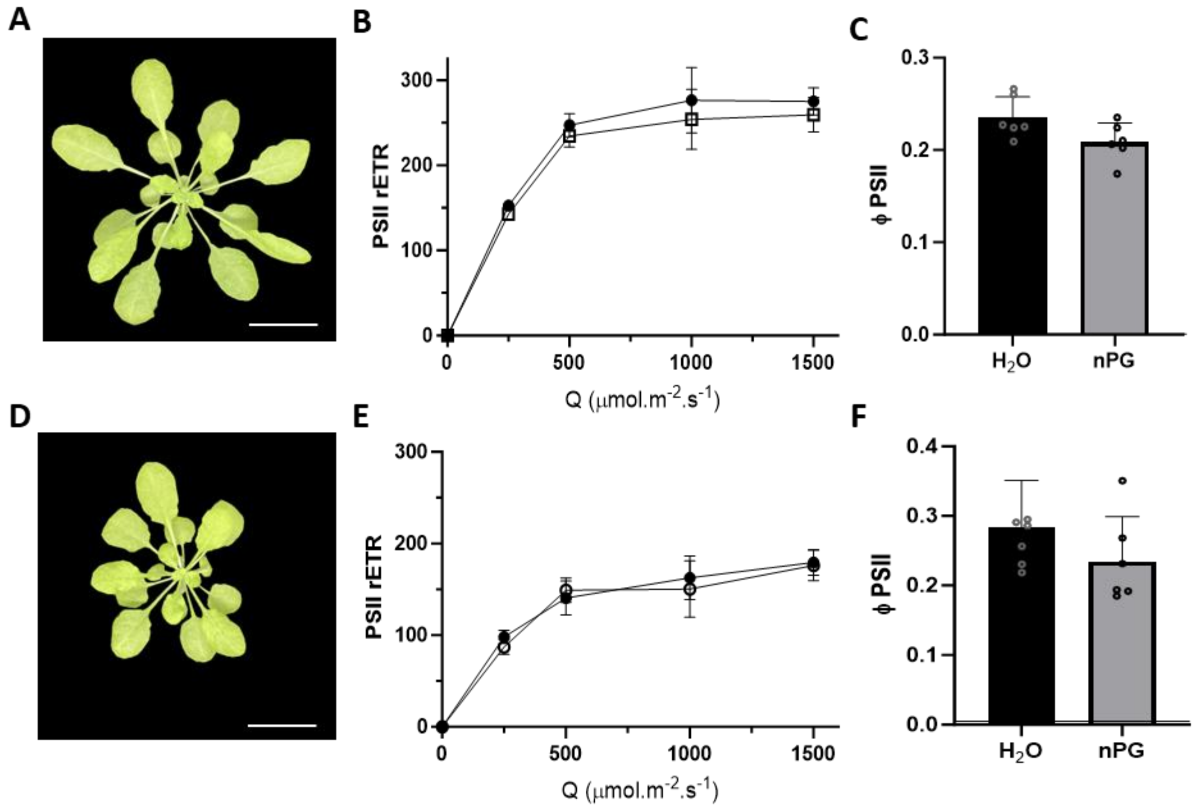

Figure S10. PTOX activity in the Arabidopsis thylakoid stacking mutants. Measurements were performed in Arabidospsis *chl1-3* (**A**-**C**) and *chl1-3xlhcb5* (**D**-**F**) genetic backgrounds. The relative electron transport rate of PSII (PSII rETR) was measured at different light intensities, and at 21% or 1% O_2 (filled and empty symbols, respectively) (**B** and **E**, n = at least 3). ΦPSII was measured in vacuum-infiltrated leaves (**C** and **F,** n = at least 6). Black bars, H_2O; grey bars, 1mM nPG. Data are the mean ± SD of independent biological replicates. White scale bar = 2 cm. All raw data, with number of replicates per data point, are provided in the source data file.

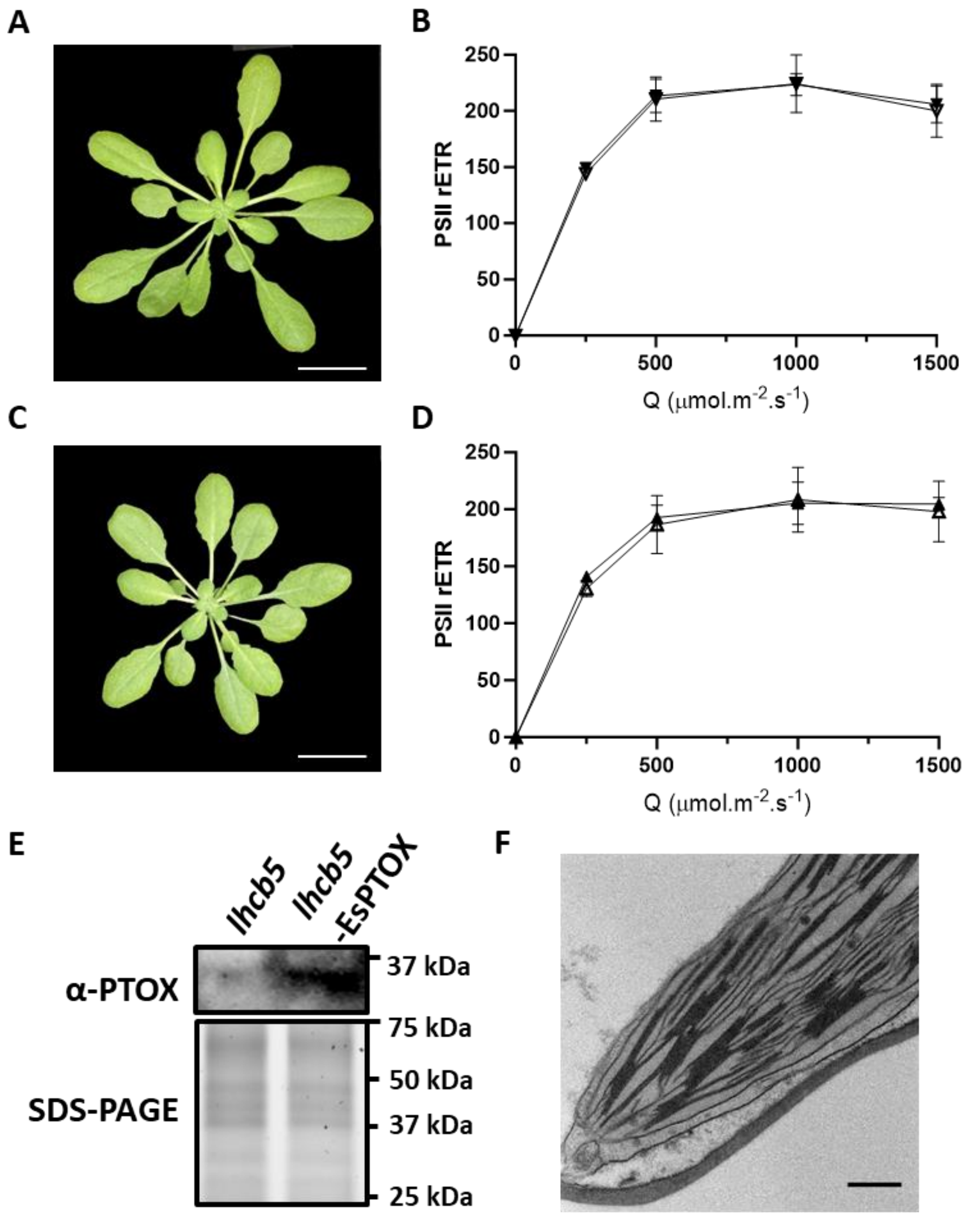

Figure S11. PTOX activity in the *lhcb5* Arabidopsis genetic background. Measurements were performed in the *lhcb5* Arabidopsis genetic background non-expressing (**A,B**) or expressing EsPTOX (**C,D**). White scale bar in **A** and **C** = 2 cm. The relative electron transport rate of PSII (PSII rETR) was measured at different light intensities, and at 21% or 1% O_2 (filled and empty symbols, respectively) (**B** and **D**). **E.** PTOX protein content in the *lhcb5* lines (loaded protein = 60 µg). Immunoblots were repeated twice, independently, obtaining the same results. **F.** Chloroplast ultrastructure of *lhcb5*-EsPTOX leaves. Scale bars = 0.5 µm**.** Data are the mean ± SD of at least 5 biological replicates. All raw data, with number of replicates per data point, are provided in the source data file.

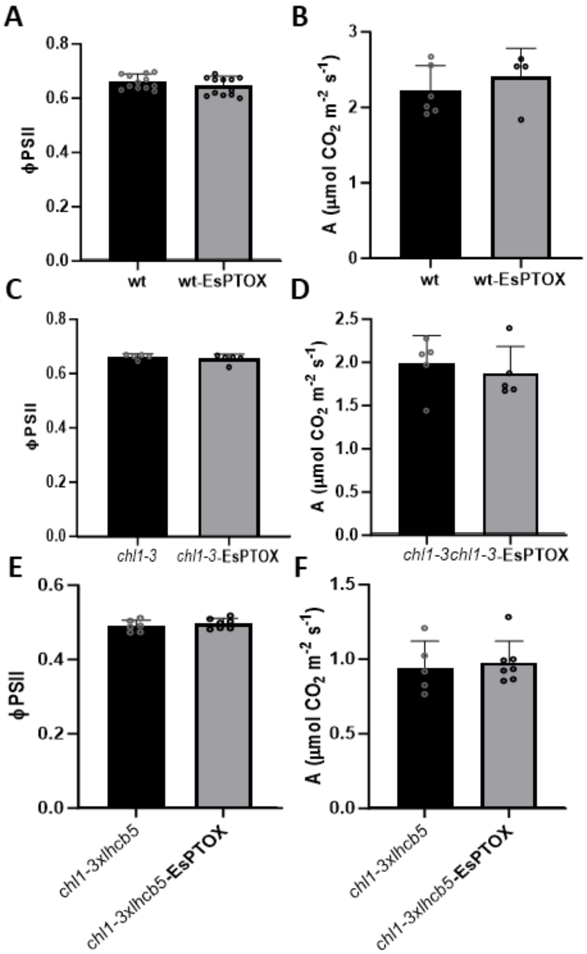

Figure S12. The effect of EsPTOX expression on photosynthetic assimilation rates in the Arabidopsis thylakoid stacking mutants under growth conditions. Quantum yield of PSII (ΦPSII) and assimilation (A, µmol $CO_2.m^{-2}.s^{-1}$) of wt and wt-EsPTOX (**A, B**), *chl1-3* and *chl1-3*-EsPTOX (**C, D**), and *chl1-3xlhcb5* and *chl1-3xlhcb5*-EsPTOX (**E, F**). Measurements were taken under growing conditions of CO_2 and light (400 CO_2 ppm and 100 µmol.$m^{-2}.s^{-1}$). Data are the mean ± SD of at least 4 biological replicates. All raw data, with number of replicates per data point, are provided in the source data file.

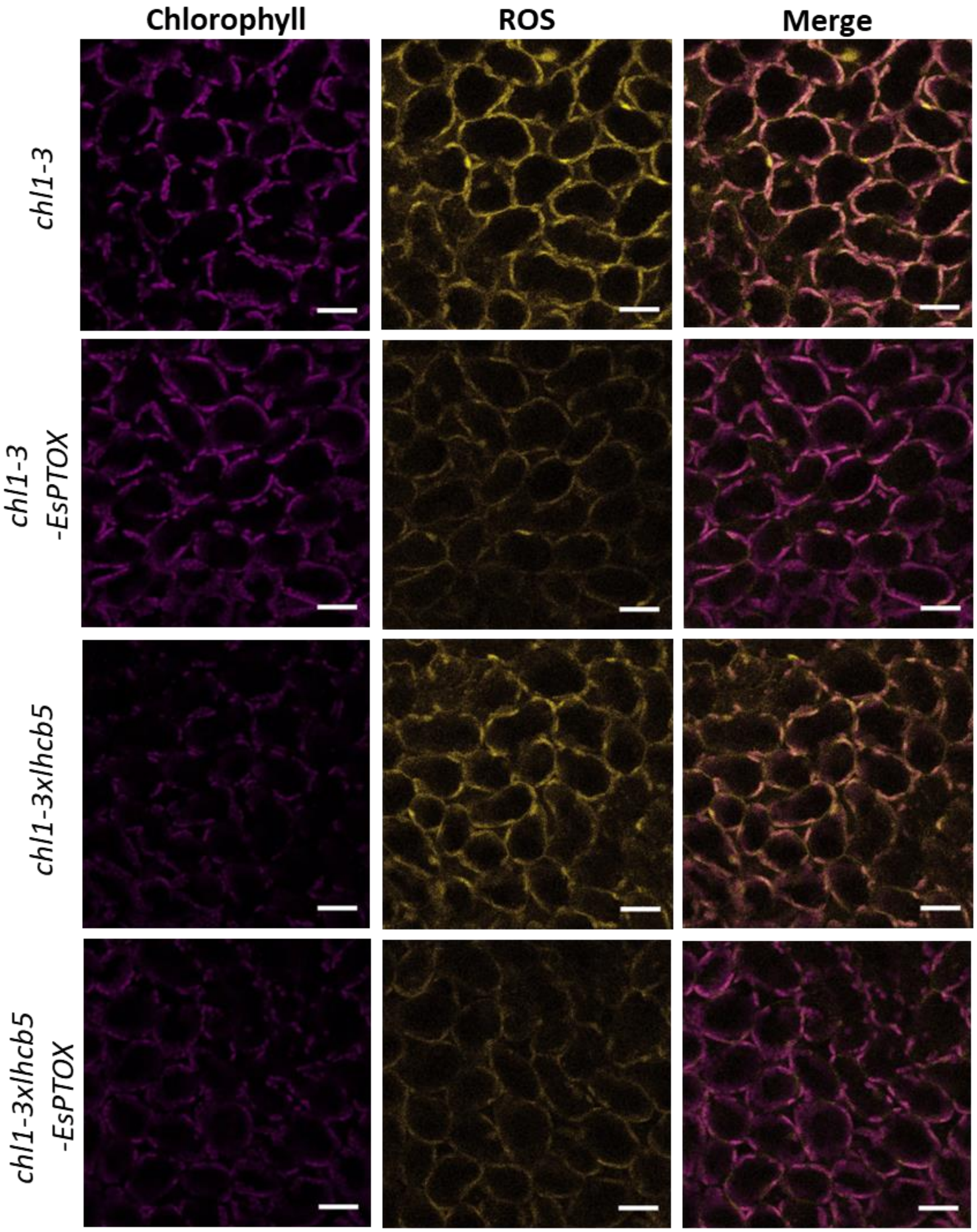

Figure S13. ROS subcellular localization in the Arabidopsis thylakoid stacking mutants under stress. Representative images from confocal laser microscopy showing subcellular localization of the DCF-DA fluorescence (yellow) and chlorophyll autofluorescence (magenta) in the *chl1-3* and *chl1-3xlhcb5* Arabidopsis genetic backgrounds subjected to stress conditions. Scale bars = 35 µm.

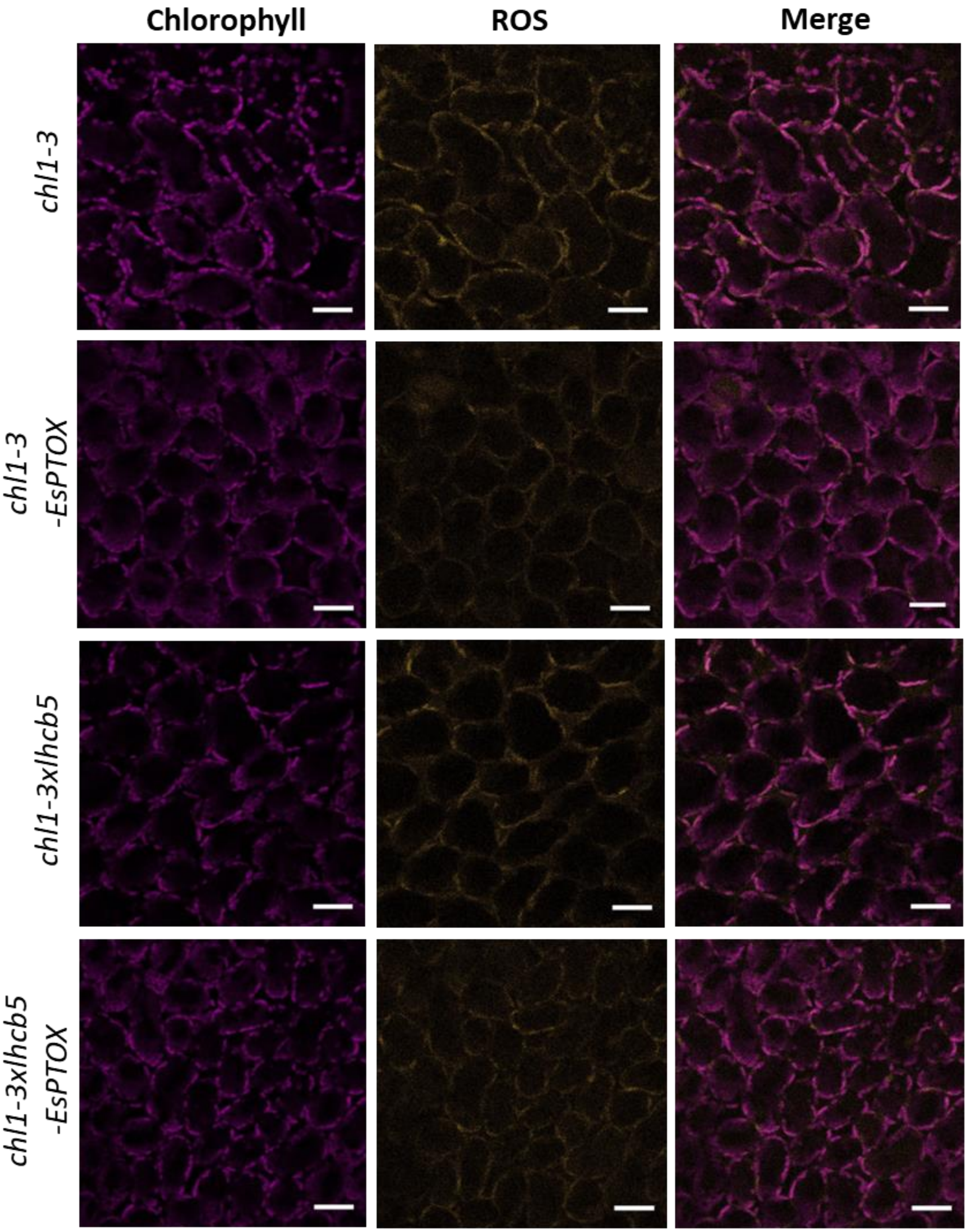

Figure S14. ROS subcellular localization in the Arabidopsis thylakoid stacking mutants under control. Representative images from confocal laser microscopy showing subcellular localization of the DCF-DA fluorescence (yellow) and chlorophyll autofluorescence (magenta), in the *chl1-3* and *chl1-3xlhcb5* Arabidopsis genetic backgrounds subjected to control conditions. Scale bars = 35 µm.

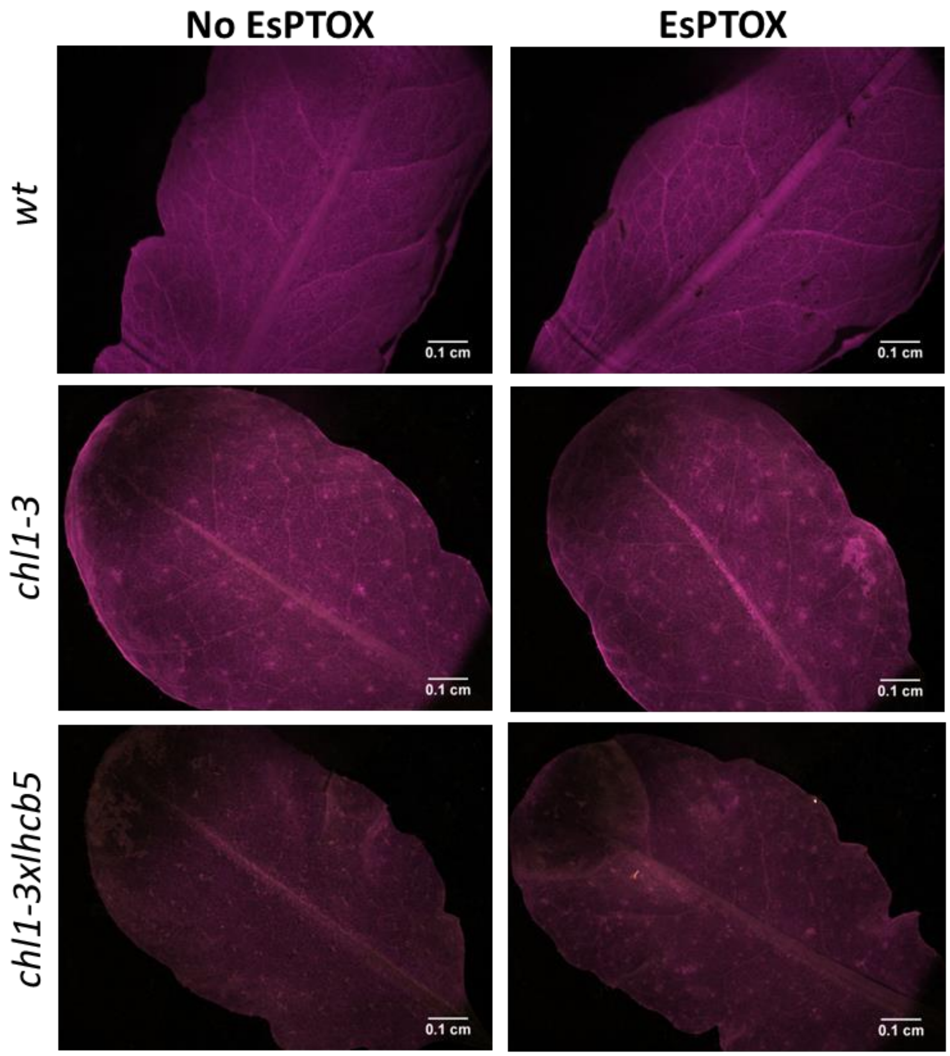

Figure S15. Absence of ROS fluorescence signal in Arabidopsis leaves not infiltrated with DFC-DA. Representative images of leaves observed under the epifluorescence microscope. Magenta fluorescence corresponds to chlorophyll autofluorescence. Scale bars = 0.1 cm.

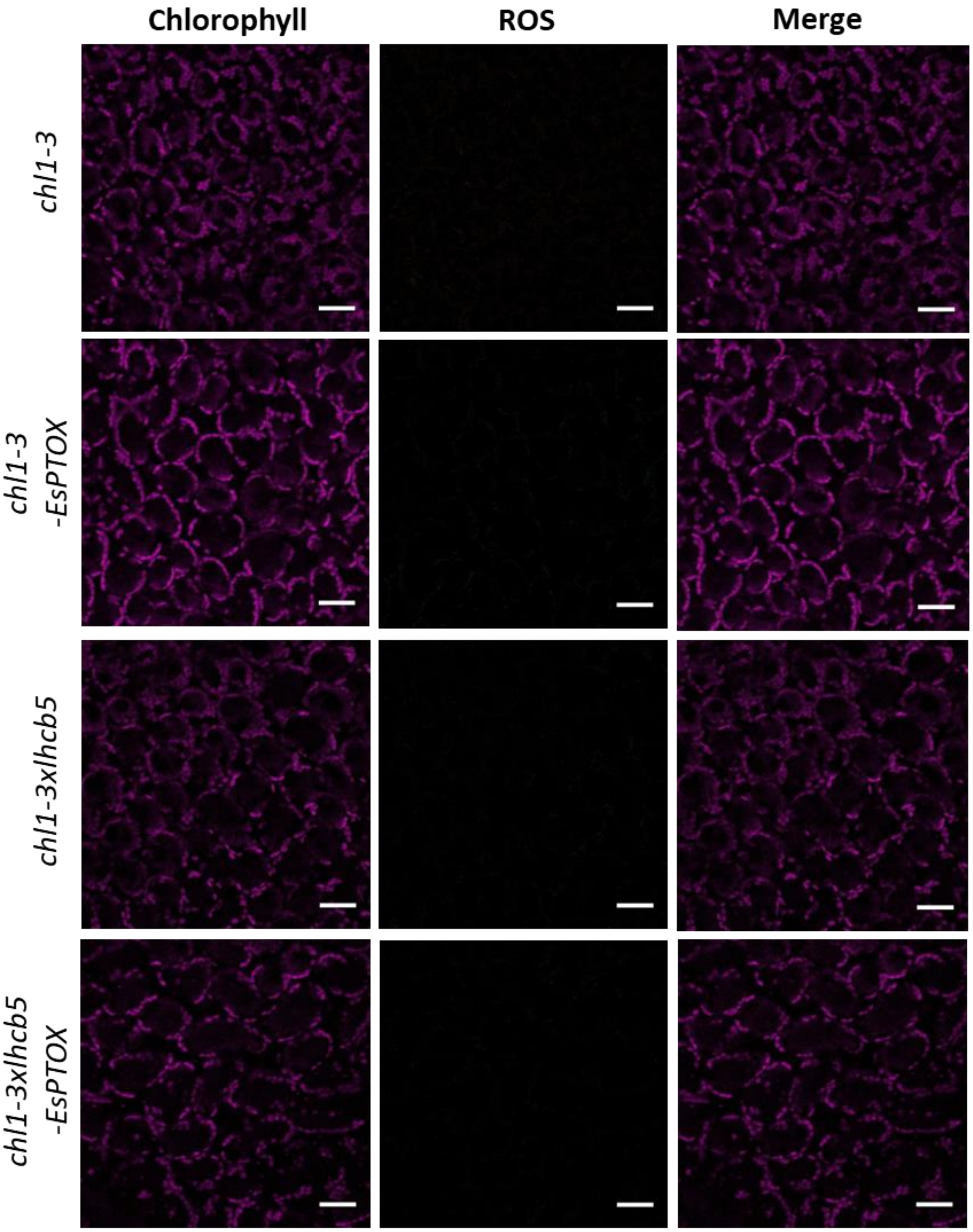

Figure S16. Absence of ROS fluorescence signals in Arabidopsis leaves not infiltrated with DFC-DA. Representative images of laser confocal microscopy of the *chl1-3* and *chl1-3xlhcb5* Arabidopsis genetic backgrounds subjected to stress conditions, but not infiltrated with DCF-DA. Magenta fluorescence corresponds to chlorophyll autofluorescence. Scale bars = 35 µm.

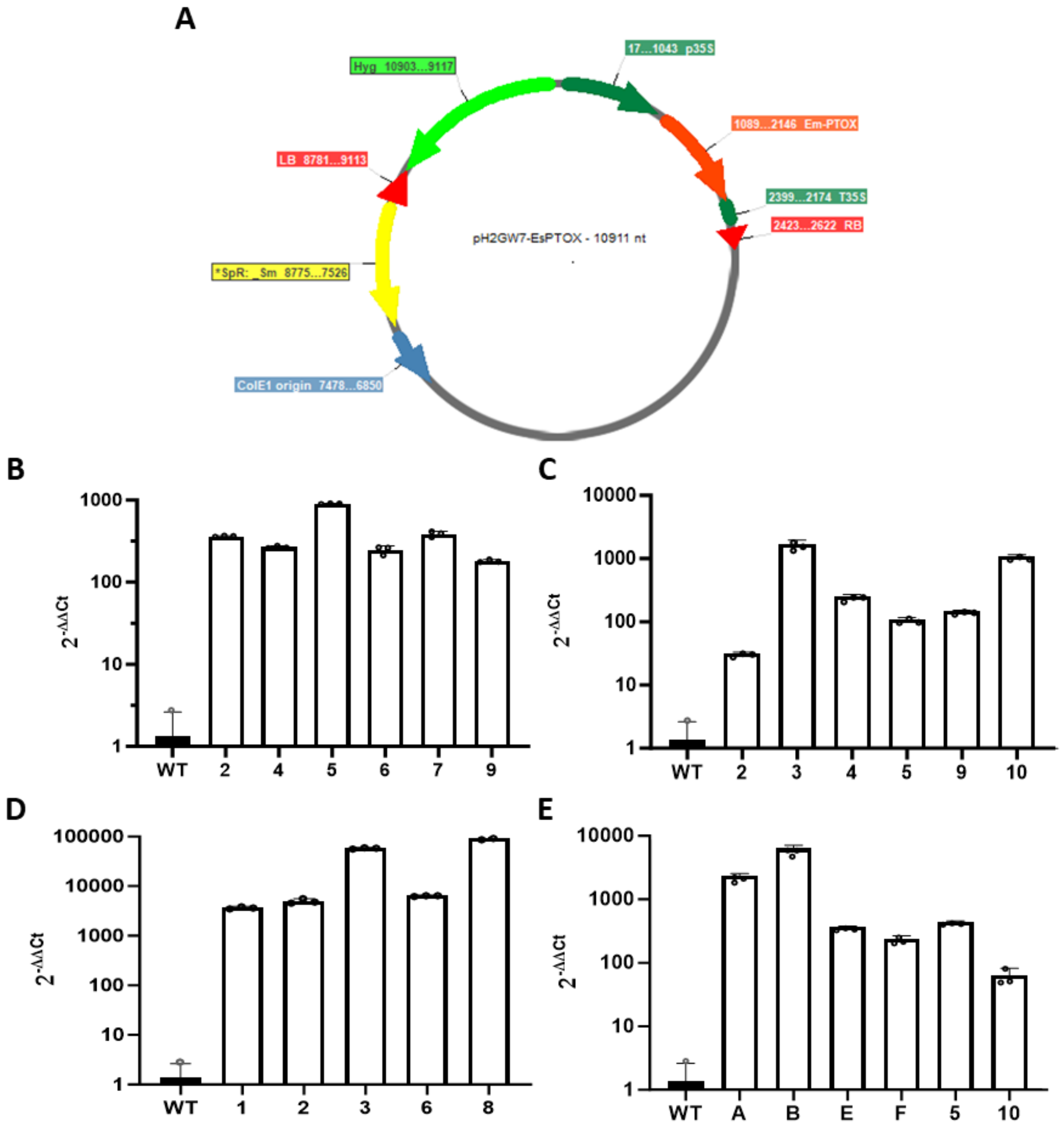

Figure S17. Transformation of *Arabidopsis thaliana* with EsPTOX. A. The pH2GW7-EsPTOX expression vector used for the transformation of Arabidopsis wt, *chl1-3*, *lhcb5* and *chl1-3xlhcb5* plants, by floral dipping. Expression of the gene was driven under control of the cauliflower mosaic virus (CaMV) 35S constitutive promoter. Hygromycin resistance gene (Hyg) was included for *in planta* selection of transgenic lines. **B-E.** EsPTOX expression levels of the different independent lines obtained from Arabidopsis wt (**B**), *lhcb5* (**C**), *chl1-3* (**D**) and *chl1-3xlhcb5* (**E**) transformation. Data are the mean ± SD of 3 biological replicates. Values were normalized to the expression levels of EsPTOX measured for the wt plants. Primers used are listed in Supp. Table 2.

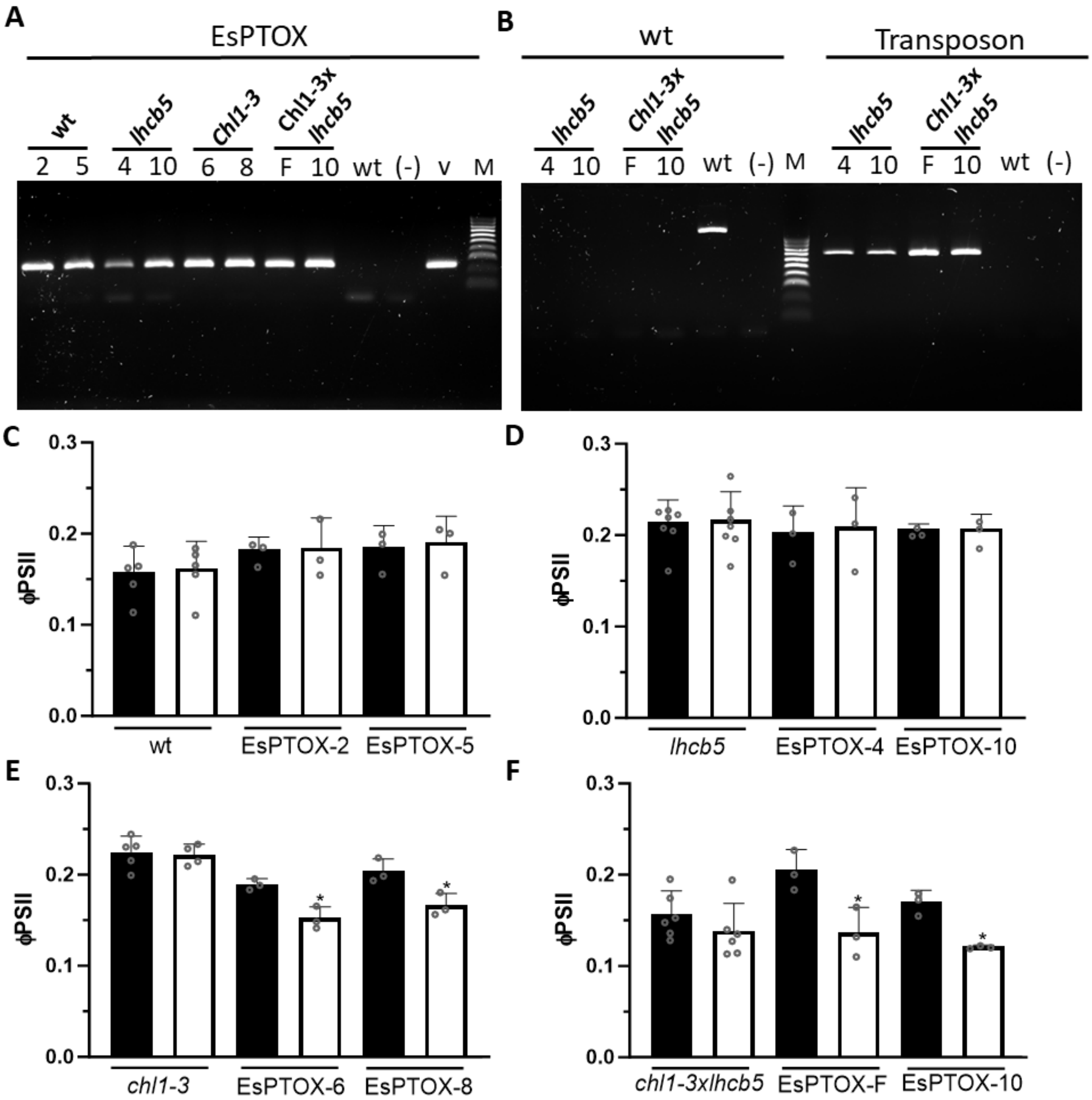

Figure S18. Genotyping and phenotyping of 2 independent EsPTOX transgenic lines, for each of the different Arabidopsis genetic backgrounds studied. PCR analysis to detect EsPTOX insertion (**A**) or homozygosis of *lhcb5* mutation (**B**). Images show representative PCR products resolved by 2% agarose gel electrophoresis. M, Molecular marker HyperladderTM100bp (Meridian Bioscience, OH, USA); v; pH2GW7-EsPTOX expression vector; (-), PCR negative control; wt, Arabidopsis Col-0. EsPTOX detection PCR was performed using the P35S-Fw and Ins-EsPTOX-Rv primers (Supp. Table 2), and an annealing temperature of 62 °C. Amplicon expected size: 254 bp. For the *lhcb5* genotyping, wt allele was identified by using the lhcb5-Fw and lhcb5-Rv primers, while the mutant allele by using the T-DNA-Fw and lhcb5-Rv primers (Supp. Table 2). In both cases the annealing temperature was 50 °C, and the expected amplicon size ≈900 and 400 bp, respectively (http://signal.salk.edu/tdnaprimers.2.html). **C-F**. Quantum yield of PSII (ΦPSII) measured at 21% or 1% O_2 (black and white bars, respectively) in the different genetic backgrounds non-expressing or expressing EsPTOX. Oxygen sensitivity of ΦPSII suggests PTOX activity. **C**, Arabidopsis wt background; **D**, *lhcb5* background; **E**, *chl1-3* background; **F**, *chl1-3xlhcb5* background. Data are the mean ± SD of at least 3 biological replicates. The asterisk indicates differences between the measurements at 21% and 1% O_2, in each case (Student's test, $p < 0.05$, two-sided). All raw data, with number of replicates per data point, are provided in the source data file.

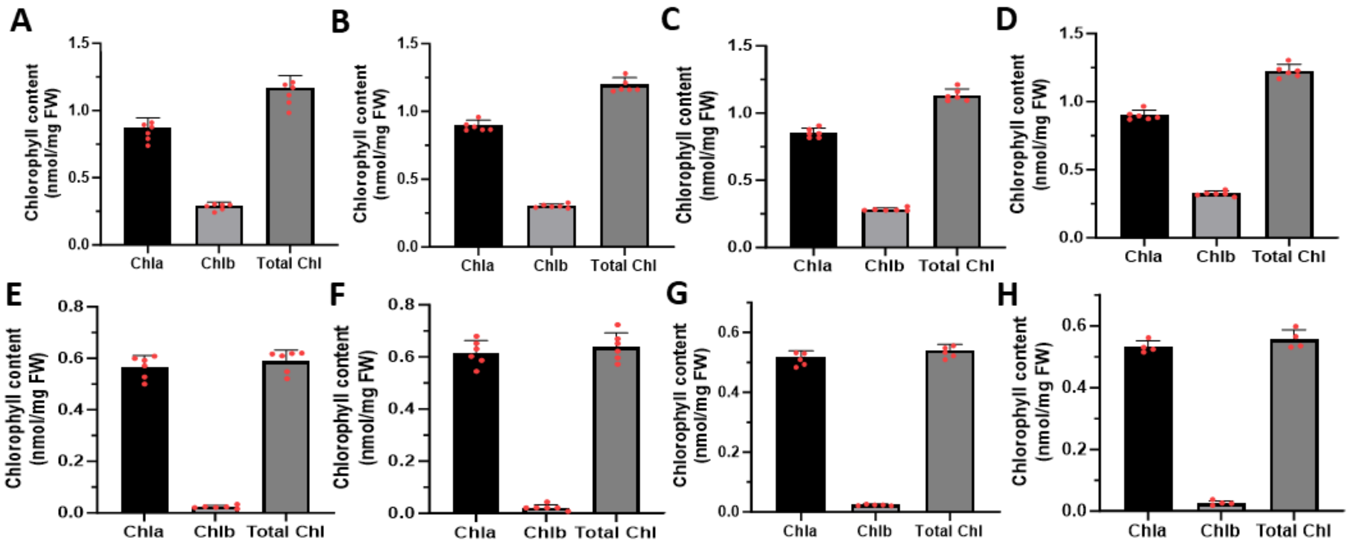

Figure S19. Chlorophyll content of the different Arabidopsis genetic background studied. Chlorophyll content (nmol.mg^{-1} FW) was measured in the Arabidopsis wt and wt-EsPTOX (**A** and **B**, respectively), *lhcb5* and *lhcb5*-EsPTOX (**C** and **D**, respectively), *chl1-3* and *chl1-3*-EsPTOX (**E** and **F**, respectively), and *chl1-3xlhcb5* and *chl1-3xlhcb5*-EsPTOX (**G** and **H**, respectively). Data are the mean ± SD of at least 4 biological replicates. All raw data, with number of replicates per data point, are provided in the source data file.

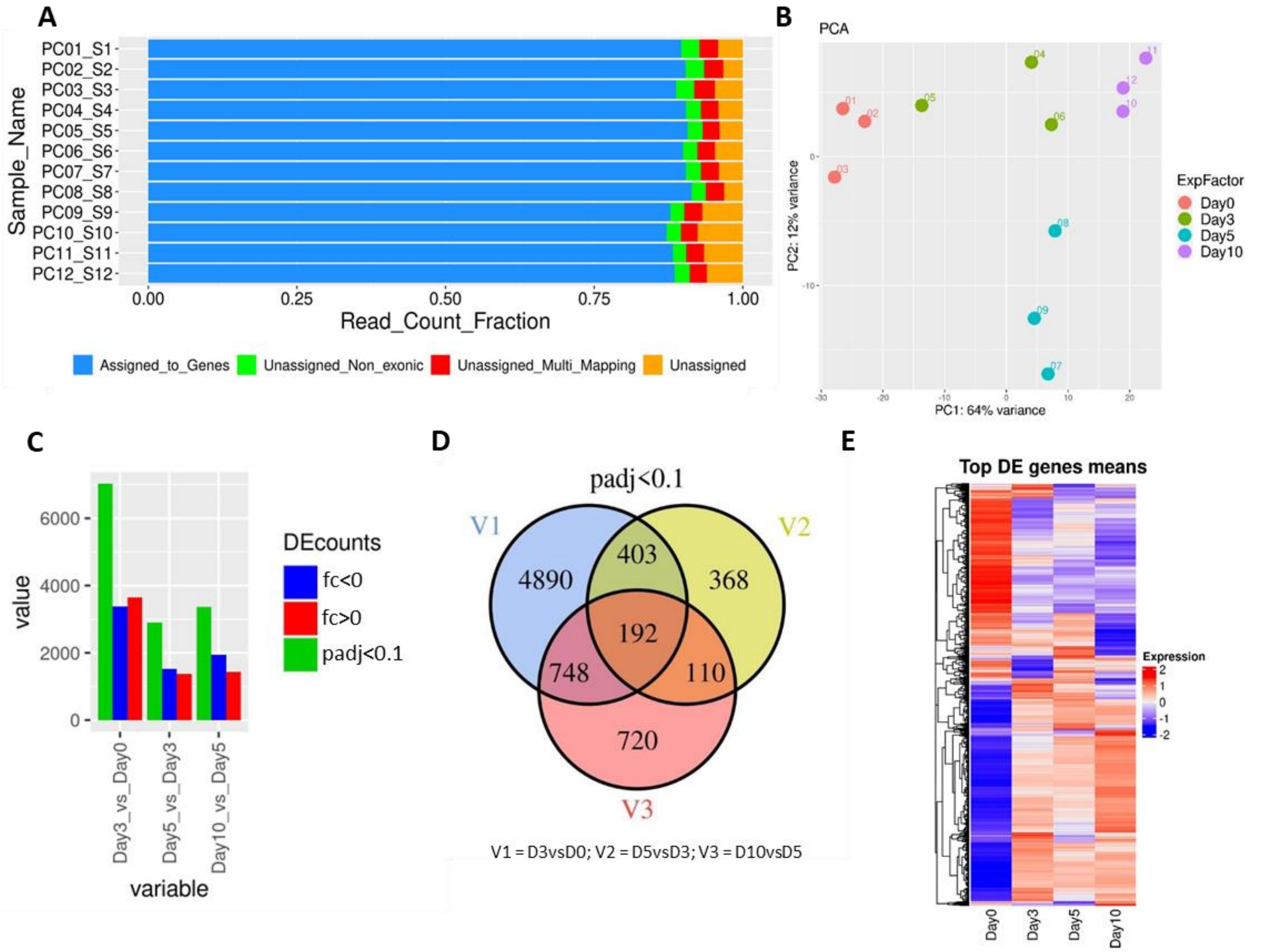

Figure S20. Time-course RNAseq analysis of *Eutrema salsugineum* plants acclimated to high light. A. Mapping and gene assignment of the total reads generated by the RNAseq, for each of the sequenced samples. **B.** Principal components analysis (PCA) plot showing the variance of the 12 sequenced samples along the high light treatment (4 time points, n = 3). **C.** Differentially expressed genes (padj < 0.1) between Day 3 and Day 0 (6,233), Day 5 and Day 3 (1,073), and Day 10 and Day 5 (1,770). From them, 2,944 and 3,289, 563 and 510, and 1,124 and 646, were down and up-regulated respectively (fc < 0; fc > 0). **D.** Venn diagram showing the differentially expressed genes (padj < 0.1) shared between the different days comparison. **E.** Hierarchical cluster of the differentially expressed genes (padj < 0.1) along the different days of the high light treatment.

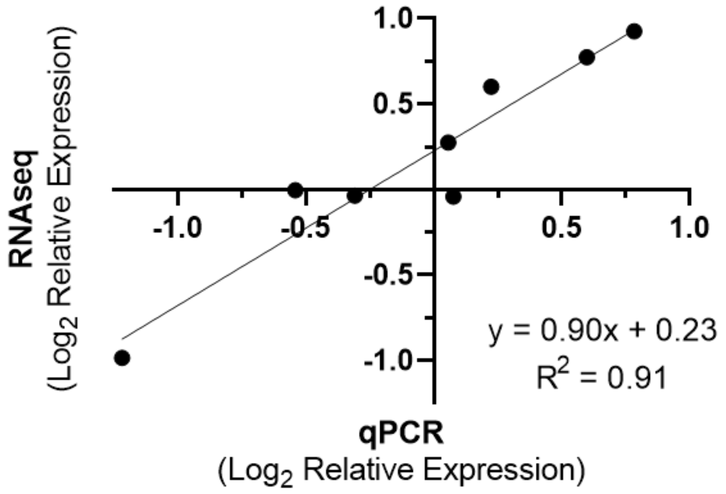

Figure S21. Time-course RNAseq validation by qPCR. Comparison of RNAseq and quantitative real-time PCR data for 3 independent genes which were classified in different expression clusters (*Thhalv10025624m* –PTOX-, *Thhalv10022631m* and *Thhalv10026385m*). Symbols represent Log_2 transformation of mean expression levels relative to day 0.

Supplementary Table 1. Annotation of the genes enriched in GO terms related to the chloroplast in the 'Cellular Compartment' category.

Classification group	Gene ID	Arabidopsis Homologue	KEGG Annotation (KO)[1]	TAIR Definition[2]	Cluster
Response to Stress	Thhalv10014567m	AT5G07020	null	MAINTENANCE OF PSII UNDER HIGH LIGHT 1 (MPH1)	2
	Thhalv10026482m	AT4G34190	null	STRESS ENHANCED PROTEIN 1 (SEP1)	2
	Thhalv10028953m	AT4G02530	null	MAINTENANCE OF PHOTOSYSTEM II UNDER HIGH LIGHT 2 (MPH2)	2
	Thhalv10016600m	AT2G39730	null	RUBISCO ACTIVASE (RCA)	2
	Thhalv10027805m	AT5G38520	null	CHLOROPHYLL DEPHYTYLASE1 (CLD1)	2
	Thhalv10008704m	AT1G34000	null	ONE-HELIX PROTEIN 2 (OHP2)	2
	Thhalv10000983m	AT5G44650	null	CHLOROPLAST PROTEIN-ENHANCING STRESS TOLERANCE (CEST)	2
	Thhalv10014048m	AT5G03880	null	THIOREDOXIN FAMILY PROTEIN, LOCATED IN CHLOROPLAST, CHANGE IN RESPONSE TO H_2O_2	2
	Thhalv10013154m	AT5G12860	null	DICARBOXYLATE TRANSPORTER 1 (DiT1)	2
	Thhalv10023228m	AT1G56500	null	SUPPRESSOR OF QUENCHING 1 (SOQ1)	2
	Thhalv10025684m	AT4G31530	null	RELAXATION OF QH (ROQH)	2
	Thhalv10023608m	AT1G54520	null	FLUCTUATING-LIGHT- ACCLIMATION PROTEIN1 (FLAP1	2
	Thhalv10006607m	AT1G01790	null	K+ EFFLUX ANTIPORTER 1 (KEA1)	2
	Thhalv10028551m	AT4G00370	null	ANION TRANSPORTER 2 (ANTR2)	2
	Thhalv10021501m	AT3G26060	K03564	THIOREDOXIN SUPERFAMILY PROTEIN	2
	Thhalv10026218m	AT4G25100	K04564	FE SUPEROXIDE DISMUTASE 1 (FSD1)	1
	Thhalv10025624m	AT4G22260	K17893	PLASTID TERMINAL OXIDASE (PTOX)	1
	Thhalv10010497m	AT3G47860	null	CHLOROPLASTIC LIPOCALIN (LCNP)	2
**Classification group**	**Gene ID**	**Arabidopsis Homologue**	**KEGG Annotation (KO)[1]**	**TAIR Definition[2]**	**Cluster**
Cyclic electron flow	Thhalv10025739m	AT4G22890	null	PGR5-LIKE A	2
	Thhalv10014691m	AT5G58260	null	NADH DEHYDROGENASE-LIKE COMPLEX N (NdhN)	2
	Thhalv10002670m	AT2G04039	null	NDHV	2
	Thhalv10005039m	AT2G01590	null	CHLORORESPIRATORY REDUCTION 3 (CRR3)	2
	Thhalv10019160m	AT1G70760	null	NADH DEHYDROGENASE-LIKE COMPLEX L (NdhL)	2

	Thhalv10002723m	AT2G05620	null	PROTON GRADIENT REGULATION 5 (PGR5)	2
	Thhalv10003269m	AT5G43750	null	PHOTOSYNTHETIC NDH SUBCOMPLEX B 5 (PnsB5)	2
	Thhalv10018808m	AT1G64770	null	PHOTOSYNTHETIC NDH SUBCOMPLEX B 2 (PnsB2)	2
	Thhalv10026209m	AT4G37925	null	NADH DEHYDROGENASE-LIKE COMPLEX M (NdhM)	2
	Thhalv10021523m	AT3G16250	null	PHOTOSYNTHETIC NDH SUBCOMPLEX B 3 (PnsB3)	2
	Thhalv10019178m	AT1G74880	null	NADH DEHYDROGENASE-LIKE COMPLEX) (NdhO)	2
	Thhalv10017187m	AT2G39470	null	PHOTOSYNTHETIC NDH SUBCOMPLEX L 1 (PnsL1)	2
	Thhalv10014707m	AT5G52780	null	PAM68-LIKE (PAM68L)	2
	Thhalv10028695m	AT4G11960	null	PGR5-LIKE B (PGRL1B)	2
	Thhalv10008902m	AT1G18730	null	PHOTOSYNTHETIC NDH SUBCOMPLEX B 4 (PnsB4)	2
**Classification group**	**Gene ID**	**Arabidopsis Homologue**	**KEGG Annotation (KO)[1]**	**TAIR Definition[2]**	**Cluster**
**Photorespiration**	Thhalv10019199m	AT5G38410	K01602	RUBISCO SMALL SUBUNIT 3B (RBCS3B)	2
	Thhalv10024934m	AT4G37930	K00600	SERINE TRANSHYDROXYMETHYLTRANSFERASE 1 (SHM1)	2
	Thhalv10027955m	AT5G38410	K01602	RUBISCO SMALL SUBUNIT 3B (RBCS3B)	2
	Thhalv10019202m	AT5G38430	K01602	RUBISCO SMALL SUBUNIT 1A (RBCS1A)	2
	Thhalv10007514m	AT1G23310	K14272	ALANINE-2-OXOGLUTARATE AMINOTRANSFERASE 1 (AOAT1)	2
	Thhalv10022711m	AT2G13360	K00830	ALANINE:GLYOXYLATE AMINOTRANSFERASE (AGT1)	2
	Thhalv10018474m	AT1G70580	K14272	ALANINE-2-OXOGLUTARATE AMINOTRANSFERASE 2 (AOAT2)	2
	Thhalv10024257m	AT4G33010	K00281	GLYCINE DECARBOXYLASE P-PROTEIN 1 (GLDP1)	2
	Thhalv10007781m	AT1G11860	K00605	AMINOMETHYLTRANSFERASE (GLDT)	2
	Thhalv10008944m	AT1G32470	K02437	GDC-H1	2
	Thhalv10017333m	AT1G32470	K02437	GDC-H1	2
	Thhalv10001891m	AT2G26080	K00281	GLYCINE DECARBOXYLASE P-PROTEIN 2 (GLDP2)	2
	Thhalv10018534m	AT1G68010	K15893	HYDROXYPYRUVATE REDUCTASE (HPR)	2

	Thhalv10020979m	AT3G14420	K11517	GLYCOLATE OXIDASE 1 (GOX1)	2
	Thhalv10020982m	AT3G14415	K11517	GLYCOLATE OXIDASE 2 (GOX2)	2
**Classification group**	**Gene ID**	**Arabidopsis Homologue**	**KEGG Annotation (KO)[1]**	**TAIR Definition[2]**	**Cluster**
CBB Cycle	Thhalv10000191m	AT2G21330	K01623	FRUCTOSE-BISPHOSPHATE ALDOLASE 1 (FBA1)	2
	Thhalv10019199m	AT5G38410	K01602	RUBISCO SMALL SUBUNIT 3B (RBCS3B)	2
	Thhalv10025379m	AT4G38970	K01623	FRUCTOSE-BISPHOSPHATE ALDOLASE 2 (FBA2)	2
	Thhalv10020621m	AT3G12780	K00927	PHOSPHOGLYCERATE KINASE 1 (PGKP1)	2
	Thhalv10021298m	AT3G04790	K01807	EMBRYO DEFECTIVE 3119 (EMB3119)	2
	Thhalv10027955m	AT5G38410	K01602	RUBISCO SMALL SUBUNIT 3B (RBCS3B)	2
	Thhalv10019202m	AT5G38430	K01602	RIBULOSE BISPHOSPHATE CARBOXYLASE SMALL CHAIN 1A (RBCS1A)	2
	Thhalv10004764m	AT3G04790	K01807	EMBRYO DEFECTIVE 3119 (EMB3119)	2
	Thhalv10021083m	AT1G13440	K00134	GLYCERALDEHYDE-3-PHOSPHATE DEHYDROGENASE C-2 (GAPC2)	1
	Thhalv10008131m	AT1G13440	K00134	GLYCERALDEHYDE-3-PHOSPHATE DEHYDROGENASE C-2 (GAPC2)	2
	Thhalv10004217m	AT1G42970	K05298	GLYCERALDEHYDE-3-PHOSPHATE DEHYDROGENASE B SUBUNIT (GAPB)	2
	Thhalv10010434m	AT3G55800	K01100	SEDOHEPTULOSE-BISPHOSPHATASE (SBPASE)	2
	Thhalv10007801m	AT1G32060	K00855	PHOSPHORIBULOKINASE (PRK)	2
	Thhalv10004349m	AT3G26650	K05298	GLYCERALDEHYDE 3-PHOSPHATE DEHYDROGENASE A SUBUNIT 1 (GAPA1)	2
**Classification group**	**Gene ID**	**Arabidopsis Homologue**	**KEGG Annotation (KO)[1]**	**TAIR Definition[2]**	**Cluster**
Protein synthesis, folding and degradation	Thhalv10026156m	AT4G19830	null	FK506-binding proteins (FKBP17-1)	2
	Thhalv10026111m	AT4G25370	null	CLP chaperone-protease (CLPT1)	2
	Thhalv10027761m	AT5G39830	null	DEG PROTEASE 8 (DEG8)	2
	Thhalv10004246m	AT3G27925	null	DEGP PROTEASE 1 (DEG1)	2
	Thhalv10024882m	AT4G17740	null	CTPA	2
	Thhalv10016805m	AT2G35260	null	BALANCE OF CHLOROPHYLL METABOLISM 1 (BCM1)	2
	Thhalv10017977m	AT2G32480	null	ARABIDOPSIS SERIN PROTEASE (ARASP)	2

	Thhalv10004302m	AT5G30510	K02945	PLASTID RIBOSOMAL PROTEIN S1 (PRPS1)	2
	Thhalv10006062m	AT3G63490	K02863	PLASTID RIBOSOMAL PROTEIN L1, PROLINE-RICH PROTEIN-LIKE 1 (PRPL1)	2
	Thhalv10004960m	AT3G27850	K02935	RIBOSOMAL PROTEIN BL12CX (BL12CX)	2
	Thhalv10019066m	AT1G78630	K02871	RIBOSOMAL PROTEIN UL13C (UL13C)	2
	Thhalv10008395m	AT1G07320	K02926	PLASTID RIBOSOMAL PROTEIN L4 (PRPL4)	2
	Thhalv10023690m	AT1G64510	K02990	PLASTID RIBOSOMAL PROTEIN OF THE 30S SUBUNIT 6 (PRPS6)	2
	Thhalv10005022m	AT5G65220	K02904	PLASTID RIBOSOMAL PROTEINS OF THE 50S SUBUNIT 29 (PRPL29)	2
	Thhalv10013384m	AT5G14590	K00031	ISOCITRATE/ISOPROPYLMALATE DEHYDROGENASE	2
	Thhalv10013566m	AT5G20070	K03426	NUDIX HYDROLASE HOMOLOG 19 (NUDX19)	2
	Thhalv10004964m	AT3G27830	K02935	RIBOSOMAL PROTEIN BL12CZ (BL12CZ)	1
	Thhalv10019289m	AT1G79850	K02961	PLASTID RIBOSOMAL SMALL SUBUNIT PROTEIN 17 (PRPS17)	1
	Thhalv10000978m	AT5G45390	K01358	CLP PROTEASE P4 (CLPP4)	1
**Classification group**	**Gene ID**	**Arabidopsis Homologue**	**KEGG Annotation (KO)[1]**	**TAIR Definition[2]**	**Cluster**
**PETC biogenesis**	Thhalv10021289m	AT3G05410	null	PHOTOSYSTEM II REACTION CENTER PSBP FAMILY PROTEIN (PPD7)	2
	Thhalv10000353m	AT2G23670	null	HOMOLOG OF SYNECHOCYSTIS (YCF37)	2
	Thhalv10013940m	AT5G54290	null	CYTOCHROME C BIOGENESIS PROTEIN FAMILY (CCDA)	2
	Thhalv10018960m	AT1G71500	null	PHOTOSYSTEM B PROTEIN 33 (PSB33)	2
	Thhalv10000289m	AT1G77090	null	PSII PSBP FAMILY PROTEIN	2
	Thhalv10002049m	AT2G20890	null	PHOTOSYSTEM II REACTION CENTER PSB29 PROTEIN (PSB29)	2
	Thhalv10021570m	AT3G17930	null	DEFECTIVE ACCUMULATION OF CYTOCHROME B6/F COMPLEX (DAC)	2
	Thhalv10008840m	AT1G05385	null	LOW PSII ACCUMULATION 19 (LPA19)	2
	Thhalv10014231m	AT5G11450	null	PSBP DOMAIN PROTEIN 5 (PPD5)	2
	Thhalv10017283m	AT2G34860	null	EMBRYO SAC DEVELOPMENT ARREST 3 (EDA3)	2
	Thhalv10002100m	AT2G27290	null	DE-ETIOLATION-INDUCED PROTEIN 1 (DEIP1)	2

	Thhalv10012349m	AT2G34860	null	EMBRYO SAC DEVELOPMENT ARREST 3 (EDA3)	2
	Thhalv10026318m	AT4G14690	null	EARLY LIGHT-INDUCIBLE PROTEIN 2 (ELIP2)	2
	Thhalv10000997m	AT5G47110	null	LIGHT-HARVESTING-LIKE 3:2 (LIL3:2)	2
	Thhalv10001663m	AT2G46820	null	PHOTOSYSTEM I P SUBUNIT (PSI-P)	2
	Thhalv10025278m	AT4G37920	null	CHLOROPLAST DEVELOPMENT AND BIOGENESIS1 (CDB1)	2
	Thhalv10028649m	AT4G01050	null	THYLAKOID RHODANESE-LIKE (TROL)	2
	Thhalv10023702m	AT1G54500	null	RBD1	2
	Thhalv10026511m	AT4G31560	null	HIGH CHLOROPHYLL FLUORESCENCE 153 (HCF153)	2
	Thhalv10015015m	AT5G51545	null	LOW PSII ACCUMULATION2 (LPA2)	2
	Thhalv10015672m	AT3G46610	null	LOW PHOTOSYNTHETIC EFFICIENCY 1 (LPE1)	2
	Thhalv10020271m	AT3G17040	null	HIGH CHLOROPHYLL FLUORESCENT 107 (HCF107)	2
	Thhalv10008318m	AT1G22700	null	PYG7 TPR-LIKE PROTEIN	2
	Thhalv10026214m	AT4G32260	K02109	PIGMENT DEFECTIVE 334 (PDE334)	2
	Thhalv10028941m	AT4G03280	K02636	PHOTOSYNTHETIC ELECTRON TRANSFER C (PETC)	2
	Thhalv10029134m	AT4G04640	K02115	GAMMA SUBUNIT OF CHLOROPLAST ATP SYNTHASE (ATPC1)	2
	Thhalv10028838m	AT4G09650	K02113	ATP SYNTHASE DELTA-SUBUNIT (ATPD)	2
	Thhalv10023930m	ATCG00280	K02705	PHOTOSYSTEM II REACTION CENTER PROTEIN C (PSBC)	2
	Thhalv10011033m	ATCG00350	K02689	PHOTOSYSTEM I REACTION CENTER (PSAA)	2

**Classification group**	**Gene ID**	**Arabidopsis Homologue**	**KEGG Annotation (KO)[1]**	**TAIR Definition[2]**	**Cluster**
**Chloroplast structural organization**	Thhalv10025758m	AT4G22240	null	FIBRILLIN 1B (FBN1B)	2
	Thhalv10004837m	AT3G26070	null	FIBRILLIN3A (FBN3A)	2
	Thhalv10016800m	AT2G35490	null	FIBRILLIN2 (FBN2)	2
	Thhalv10028808m	AT4G04020	null	FIBRILLIN 1A (FIB1A)	2
	Thhalv10021276m	AT3G23400	null	FIBRILLIN4 (FBN4)	2
	Thhalv10011532m	AT1G51110	null	FIBRILLIN10 (FBN10)	2
	Thhalv10021454m	AT3G07430	null	YGGT FAMILY PROTEIN (YLMG1-1)	1

	Thhalv10019896m	AT3G18110	null	ALBINO COTYLEDON MUTANT1 (ACM1)	1
	Thhalv10005036m	AT1G42960	null	CARBOXYLTRANSFERASE INTERACTOR1 (CTI1)	2
	Thhalv10020179m	AT3G07700	null	SALT-INDUCED ABC1 KINASE 7 (ABC1K7)	2
	Thhalv10027870m	AT5G38660	null	ACCLIMATION OF PHOTOSYNTHESIS TO ENVIRONMENT (APE1)	2
	Thhalv10014904m	AT5G08050	null	RIQ1	2
	Thhalv10019168m	AT1G74730	null	RIQ2	2
	Thhalv10029019m	AT4G01150	null	CURVATURE THYLAKOID 1A (CURT1A)	2
	Thhalv10026263m	AT4G13200	null	PASTOGLOBULAR PROTEIN 18 (PG18)	2
	Thhalv10020153m	AT3G16000	null	MAR BINDING FILAMENT-LIKE PROTEIN 1 (MFP1)	2
	Thhalv10006729m	AT1G03160	null	FZO-LIKE (FZL)	2

**Classification group**	**Gene ID**	**Arabidopsis Homologue**	**KEGG Annotation (KO)[1]**	**TAIR Definition[2]**	**Cluster**
**Translocation and transport related proteins**	Thhalv10004017m	AT5G62810	K13343	PEROXISOME DEFECTIVE 2 (PEX14)	2
	Thhalv10004795m	AT5G23040	null	CHAPERONE-LIKE PROTEIN OF POR1 (CPP1)	2
	Thhalv10014662m	AT5G55710	null	TRANSLOCON AT THE INNER ENVELOPE MEMBRANE OF CHLOROPLASTS 20-V (TIC20-V)	2
	Thhalv10014513m	AT5G52440	null	HIGH CHLOROPHYLL FLUORESCENCE 106 (HCF106)	2
	Thhalv10000343m	AT2G24020	null	SUPPRESSOR OF TIC40 2 (STIC2)	2
	Thhalv10013528m	AT5G16620	null	TRANSLOCON AT THE INNER ENVELOPE MEMBRANE OF CHLOROPLASTS 40 (TIC40)	2
	Thhalv10020309m	AT3G18890	null	TRANSLOCON AT THE INNER ENVELOPE MEMBRANE OF CHLOROPLASTS 62 (TIC62)	2
	Thhalv10018914m	AT1G72640	null	NAD(P)-BINDING ROSSMANN-FOLD SUPERFAMILY PROTEIN	2
	Thhalv10025732m	AT4G23430	null	TRANSLOCON AT THE INNER ENVELOPE MEMBRANE OF CHLOROPLASTS 32-IVA (TIC32-IVA)	2
	Thhalv10013251m	AT5G01590	null	TRANSLOCON AT THE INNER ENVELOPE MEMBRANE OF CHLOROPLASTS 56 (TIC56)	2

	Thhalv10014315m	AT5G14100	null	ARABIDOPSIS THALIANANON-INTRINSIC ABC PROTEIN 14 (ABCI11)	2
	Thhalv10022775m	AT2G15290	null	TRANSLOCON AT INNER MEMBRANE OF CHLOROPLASTS 21 (TIC21)	2
	Thhalv10025564m	AT4G13590	null	CHLOROPLAST MANGANESE TRANSPORTER1 (CMT1)	2
	Thhalv10025967m	AT4G33460	null	ATP-BINDING CASSETTE I10 (ATNAP13)	2
	Thhalv10026464m	AT4G38490	null	SECE2	2
	Thhalv10011270m	AT1G54350	null	ATP-BINDING CASSETTE D2 (ABCD2)	2
	Thhalv10020028m	AT3G16290	null	EMBRYO DEFECTIVE 2083 (EMB2083)	1
	Thhalv10012645m	AT5G22640	null	TRANSLOCON AT THE INNER ENVELOPE MEMBRANE OF CHLOROPLASTS 100 (TIC100)	1
	Thhalv10022631m	AT2G18710	K10956	SECY HOMOLOG 1 (SCY1)	2
	Thhalv10024543m	AT4G24280	K03283	HEAT SHOCK PROTEIN 70-6 (HSP70-6)	2
	Thhalv10024544m	AT4G24280	K03283	HEAT SHOCK PROTEIN 70-6 (HSP70-6)	1
	Thhalv10008025m	AT1G06870	K03100	PLASTIDIC TYPE I SIGNAL PEPTIDASE 2A (PLSP2A)	1
	Thhalv10007427m	AT1G24490	K03217	ALBINA 4 (ALB4)	1
	Thhalv10000946m	AT2G01110	K03118	TWIN-ARGININE TRANSLOCATION C (TATC)	2
	Thhalv10001508m	AT2G45770	K03110	ALPHA SUBUNIT CHLOROPLAST SRP RECEPTOR HOMOLOG (CPFTSY)	2
	Thhalv10005085m	AT5G28750	K03116	TWIN-ARGININE TRANSLOCATION A (TATA)	2

[1] https://www.genome.jp/kegg/
[2] https://www.arabidopsis.org/

Supplementary Table 2. Primers used for genotyping and qRT-PCR

	Primers	Sequence
**Genotyping**	P35S-Fw	5’-CGC ACA ATC CCA CTA TCC TT-3’
	Ins-EsPTOX-Rv	5’-GCG TCG AAG AGC AAT CAA AG-3’
	lhcb5-Fw	5’-CGC CAC TAG TGA TAA AAT CGC-3’
	lhcb5-Rv	5’-TAA ACG GTG AAG TTG CTG GAG-3’
	T-DNA-Fw (LBb1.3)	5’-ATT TTG CCG ATT TCG GAA C-3’
**qRT-PCR**	q-EsPTOX-234F	5’-GCC GCC GTT TCG TAC AGT A-3’
	q-EsPTOX-302R	5’-GAG CAG GCG AGG AGA AGA GA-3’
	Thhalv10022631m-Fw	5’-TCGATCCCTCTAGTCCGACC-3’
	Thhalv10022631m-Rv	5’-AGAACGGCCAGAAATGCAGA-3’
	Thhalv10026385m-Fw	5’-ACCACCGATGAACACAACCT-3’
	Thhalv10026385m-Rv	5’-GATCTCACTGGAGGCCAACC-3’
	Em-GADPH-Fw	5’-AAG TTC TCG CTC AAC GCA AT-3’
	Em-GADPH-Rv	5’-TTG GTG ACA GCA GGT CGA G-3’
	At-GADPH-Fw	5’-AAA CTT GTC GCT CAA TGC AAT C-3’
	At-GADPH-Rv	5’-TTG GTG ACA ACA GGT CAA GCA-3’
	PsbA-Em-Fw	5’-CGC ATA CCC AGA CGG AAA CT-3’
	PsbA-Em-Rv	5’-CGC ATT CAT TGC TGC TCC TC-3’

SI References

1- Armenteros, J. J. A., Salvatore, M., Emanuelsson, O., Winther, O., Von Heijne, G., Elofsson, A., & Nielsen, H. (2019). Detecting sequence signals in targeting peptides using deep learning. *Life science alliance*, *2*(5).

2- Petsalaki, E. I., Bagos, P. G., Litou, Z. I., & Hamodrakas, S. J. (2006). PredSL: a tool for the N-terminal sequence-based prediction of protein subcellular localization. *Genomics, Proteomics & Bioinformatics*, *4*(1), 48-55.

3- Mori, H., & Cline, K. (2001). Post-translational protein translocation into thylakoids by the Sec and ΔpH-dependent pathways. *Biochimica et Biophysica Acta (BBA)-Molecular Cell Research*, *1541*(1-2), 80-90.

4- Dittmar, J., Schlesier, R., & Klösgen, R. B. (2014). Tat transport of a Sec passenger leads to both completely translocated as well as membrane-arrested passenger proteins. Biochimica et Biophysica Acta (BBA)-Molecular Cell Research, 1843(2), 446-453.
