# Supplementary material for: Proximity to Photosystem II is necessary for activation of Plastid Terminal Oxidase (PTOX) for photoprotection
Source: Nat Commun. 2024 Jan 4;15:287. doi: 10.1038/s41467-023-44454-x (PMC10767095; doi:10.1038/s41467-023-44454-x)
Supplement: Supplementary file 3 — Reporting Summary [file 41467_2023_44454_MOESM3_ESM.pdf]

nature portfolio

Corresponding author(s): Prof. Giles N. Johnson

Last updated by author(s): Oct 20, 2023

Reporting Summary

Nature Portfolio wishes to improve the reproducibility of the work that we publish. This form provides structure for consistency and transparency in reporting. For further information on Nature Portfolio policies, see our Editorial Policies and the Editorial Policy Checklist.

Statistics

For all statistical analyses, confirm that the following items are present in the figure legend, table legend, main text, or Methods section.

n/a	Confirmed	
☐	☒	The exact sample size (*n*) for each experimental group/condition, given as a discrete number and unit of measurement
☐	☒	A statement on whether measurements were taken from distinct samples or whether the same sample was measured repeatedly
☐	☒	The statistical test(s) used AND whether they are one- or two-sided<br>*Only common tests should be described solely by name; describe more complex techniques in the Methods section.*
☒	☐	A description of all covariates tested
☐	☒	A description of any assumptions or corrections, such as tests of normality and adjustment for multiple comparisons
☐	☒	A full description of the statistical parameters including central tendency (e.g. means) or other basic estimates (e.g. regression coefficient) AND variation (e.g. standard deviation) or associated estimates of uncertainty (e.g. confidence intervals)
☒	☐	For null hypothesis testing, the test statistic (e.g. *F*, *t*, *r*) with confidence intervals, effect sizes, degrees of freedom and *P* value noted<br>*Give P values as exact values whenever suitable.*
☒	☐	For Bayesian analysis, information on the choice of priors and Markov chain Monte Carlo settings
☒	☐	For hierarchical and complex designs, identification of the appropriate level for tests and full reporting of outcomes
☒	☐	Estimates of effect sizes (e.g. Cohen's *d*, Pearson's *r*), indicating how they were calculated

Our web collection on statistics for biologists contains articles on many of the points above.

Software and code

Policy information about availability of computer code

Data collection

Leica LAS AF LITE Software (Leica Microsystems CMS GmbH, Germany) - Confocal and modular stereo fluorescence microscope images collection.
Talos L120C TEM Software (ThermoFisher Scientific, MA, USA) - TEM images collection.
LI-6400XT Infrared Gas Analyzer Software (LI-COR, NE, USA) - Gas exchange data collection.
PAM-101 fluorometer (Heinz Walz, GmbH, Germany) and customized software designed by GNJ - Chlorophyll a fluorescence data collection.
Chemidoc MP System (Bio-RAD, USA) - Western blot data collection.
StepOnePlus Real-Time PCR System Software (Thermo Fisher Scientific, MA, USA) - qPCR Data collection.
RNAseq Data Collection and processing Softwares:
FastQC (http://www.bioinformatics.babraham.ac.uk/projects/fastqc/); Trimmomatic_0.39; STAR_2.7.7a

Data analysis

GraphPad Software (Version 10.0.02-232, CA, USA) - Graphs and statistica analysis
FIJI Image J Software (National Health Institute, MD, USA) - Image analysis
StepOne Software v2.3 (Thermo Fisher Scientific, USA) - qPCR data analysis
RNAseq Data analysis:
DESeq2_1.18.1; kmeans (R version 4.2.0); Gene Ontology (GO) Enrichment Analysis (GO released date 2022-09-19); ClusterProfiler v4.4.4

For manuscripts utilizing custom algorithms or software that are central to the research but not yet described in published literature, software must be made available to editors and reviewers. We strongly encourage code deposition in a community repository (e.g. GitHub). See the Nature Portfolio guidelines for submitting code & software for further information.

Data

Policy information about availability of data

All manuscripts must include a data availability statement. This statement should provide the following information, where applicable:

- Accession codes, unique identifiers, or web links for publicly available datasets
- A description of any restrictions on data availability
- For clinical datasets or third party data, please ensure that the statement adheres to our policy

Illumina reads generated during the Time-Course RNAseq of Eutrema salsugineum plants acclimated to high light are available in BioStudies of the EMBL-EBI (https://www.ebi.ac.uk/biostudies/), under the accession number E-MTAB-12913. Original electron microscopy and confocal images are available on request to the corresponding author. All other data generated or analyzed during this study are included in this published article (and its supplementary information files).

Research involving human participants, their data, or biological material

Policy information about studies with human participants or human data. See also policy information about sex, gender (identity/presentation), and sexual orientation and race, ethnicity and racism.

Reporting on sex and gender	*Use the terms sex (biological attribute) and gender (shaped by social and cultural circumstances) carefully in order to avoid confusing both terms. Indicate if findings apply to only one sex or gender; describe whether sex and gender were considered in study design; whether sex and/or gender was determined based on self-reporting or assigned and methods used. Provide in the source data disaggregated sex and gender data, where this information has been collected, and if consent has been obtained for sharing of individual-level data; provide overall numbers in this Reporting Summary. Please state if this information has not been collected. Report sex- and gender-based analyses where performed, justify reasons for lack of sex- and gender-based analysis.*
Reporting on race, ethnicity, or other socially relevant groupings	*Please specify the socially constructed or socially relevant categorization variable(s) used in your manuscript and explain why they were used. Please note that such variables should not be used as proxies for other socially constructed/relevant variables (for example, race or ethnicity should not be used as a proxy for socioeconomic status). Provide clear definitions of the relevant terms used, how they were provided (by the participants/respondents, the researchers, or third parties), and the method(s) used to classify people into the different categories (e.g. self-report, census or administrative data, social media data, etc.) Please provide details about how you controlled for confounding variables in your analyses.*
Population characteristics	*Describe the covariate-relevant population characteristics of the human research participants (e.g. age, genotypic information, past and current diagnosis and treatment categories). If you filled out the behavioural & social sciences study design questions and have nothing to add here, write "See above."*
Recruitment	*Describe how participants were recruited. Outline any potential self-selection bias or other biases that may be present and how these are likely to impact results.*
Ethics oversight	*Identify the organization(s) that approved the study protocol.*

Note that full information on the approval of the study protocol must also be provided in the manuscript.

Field-specific reporting

Please select the one below that is the best fit for your research. If you are not sure, read the appropriate sections before making your selection.

☒ Life sciences ☐ Behavioural & social sciences ☐ Ecological, evolutionary & environmental sciences

For a reference copy of the document with all sections, see nature.com/documents/nr-reporting-summary-flat.pdf

Life sciences study design

All studies must disclose on these points even when the disclosure is negative.

Sample size	No sample-size calculation was performed. Sample size was defined based on the minimal number of biological replicates needed to perform statistics (n = 3), being this value increased depending on the variability of the measurements. In the case of the ultrastructure parameters, we defined the sample size based on examples in the literature where similar measurements were performed.
Data exclusions	If the variability of the samples was high, only when biological replicates were n =8 or higher, we considered excluding 10 % of the highest and lowest values, respectively, and at the same time. When clear outliers of the data sets were observed, these were also considered for exclusion.
Replication	Experiments were performed at least twice with independent biological replicates.
Randomization	Genotypes were placed randomly in the different trays exposed to control or high light conditions.

Blinding	Blinding was not possible since the phenotype of the plants exposed to high light and control conditions were different.

Reporting for specific materials, systems and methods

We require information from authors about some types of materials, experimental systems and methods used in many studies. Here, indicate whether each material, system or method listed is relevant to your study. If you are not sure if a list item applies to your research, read the appropriate section before selecting a response.

Materials & experimental systems

n/a	Involved in the study	
☐	☒	Antibodies
☒	☐	Eukaryotic cell lines
☒	☐	Palaeontology and archaeology
☒	☐	Animals and other organisms
☒	☐	Clinical data
☒	☐	Dual use research of concern
☐	☒	Plants

Methods

n/a	Involved in the study	
☒	☐	ChIP-seq
☒	☐	Flow cytometry
☒	☐	MRI-based neuroimaging

Antibodies

Antibodies used	PTOX antibody (AS16 3692, Agrisera); HRP-conjugated secondary antibody (anti-rabbit IgG, AS09 602, Agrisear)
Validation	PTOX was validated in Arabidopsis by the supplier (please see references below), and was tested in Eutrema salsugineum before (Stepien and Johnson, 2018). Manufacturer states cross reactivity to 52 KDa band.<br><br>Stepien, P., & Johnson, G. N. (2018). Plastid terminal oxidase requires translocation to the grana stacks to act as a sink for electron transport. Proceedings of the National Academy of Sciences, 115(38), 9634-9639.<br>Urban, Rogowski & Romanowska (2022), Crucial role of the PTOX and CET pathways in optimizing ATP synthesis in mesophyll chloroplasts of C3 and C4 plants, Environmental and Experimental Botany, Volume 202, October 2022, 105024, https://doi.org/10.1016/j.envexpbot.2022.105024<br>Pralon et al. (2020). Mutation of the Atypical Kinase ABC1K3 Partially Rescues the PROTON GRADIENT REGULATION 6 Phenotype in Arabidopsis thaliana. Front. Plant Sci., 25 March 2020<br>Bolte et al. (2020). Dynamics of the localization of the plastid terminal oxidase PTOX inside the chloroplast. J Exp Bot. 2020 Feb 15. pii: eraa074. doi: 10.1093/jxb/eraa074.<br>Cournac et al. (2000b). Flexibility in photosynthetic electron transport: a newly identified chloroplast oxidase involved in chlororespiration. Philos Trans R Soc Lond B Biol Sci. 2000 Oct 29;355(1402):1447-54<br>Cournac et al. (2000a). Electron flow between photosystem II and oxygen in chloroplasts of photosystem I-deficient algae is mediated by a quinol oxidase involved in chlororespiration. J Biol Chem. 2000 Jun 9;275(23):17256-62.

Dual use research of concern

Policy information about dual use research of concern

Hazards

Could the accidental, deliberate or reckless misuse of agents or technologies generated in the work, or the application of information presented in the manuscript, pose a threat to:

No	Yes	
☐	☐	Public health
☐	☐	National security
☐	☐	Crops and/or livestock
☐	☐	Ecosystems
☐	☐	Any other significant area

Experiments of concern

Does the work involve any of these experiments of concern:

No	Yes	
☐	☐	Demonstrate how to render a vaccine ineffective
☐	☐	Confer resistance to therapeutically useful antibiotics or antiviral agents
☐	☐	Enhance the virulence of a pathogen or render a nonpathogen virulent
☐	☐	Increase transmissibility of a pathogen
☐	☐	Alter the host range of a pathogen
☐	☐	Enable evasion of diagnostic/detection modalities
☐	☐	Enable the weaponization of a biological agent or toxin
☐	☐	Any other potentially harmful combination of experiments and agents

Plants

Seed stocks	chl1-3 seeds were kindly given by Dr. Krishna Niyogi but are also available from NASC (https://arabidopsis.info/BasicForm; ID: N3121), while lhcb5 seeds were obtained from NASC seeds collection (ID: N656198).
Novel plant genotypes	The chl1-3xlhcb5 was obtained by crossing homozygous lhcb5 and chl1-3 plants. Transgenic plants expressing the PTOX gene from E. salsugineum (EsPTOX) were generated by transformation. The pH2GW7-EsPTOX expression vector was introduced into Agrobacterium tumefaciens strain GV3101, and grown in medium supplemented with antibiotics to select for positive colonies. PCR-verified Agrobacterium colonies were used for transformation of Arabidopsis wt, chl1-3, lhcb5 and chl1-3xlhcb5, by floral dipping
Authentication	Plants were left to flower and seeds were harvested, surface-sterilized and sowed in half-strength Murashige and Skoog (MS) selective medium (50 mg.L-1 hygromycin) for the selection of transgenic lines. Long roots seedlings were selected and transferred to 7.5-cm pots filled with Levington F2 compost (Levington Advance, UK). Presence of the EsPTOX gene was confirmed by PCR, and its expression estimated by quantitative PCR. Homozygosity for the lhcb5 backgrounds was checked by PCR, while homozygosity for chl1-3 was checked by chlorophyll extraction. Two independent EsPTOX expressor lines were selected for each genetic background and phenotyped using rETR oxygen sensitivity (see Measurements of PTOX activity). Since the EsPTOX lines in each genetic background showed a similar behavior, only one line was selected in each case for further experiments (particularly, wt-EsPTOX-5; lhcb5-EsPTOX-4; chl1-3-EsPTOX-6 and chl1-3xlhcb5-EsPTOX-F).
